# Effects of refined carbohydrates on immune markers after weight loss on a very-low-carbohydrate diet: A randomized controlled feeding trial

**DOI:** 10.1016/j.cpblue.2026.100122

**Published:** 2026-09-09

**Authors:** Ezequiel Dantas, Yushu Shi, Jeshua Kim, Anirudh Murthy, Alexander Emmanuelli, Shiri Li, Tiffany Perrier, Tanvir Ahmed, Isaac Nathoo, Carolina E. Echeverria, Yingzhao Wang, Omar Abdallah, Tobias Janowitz, Maurice A. Hurd, Noriko Suzuki-Kemuriyama, Cara B. Ebbeling, David B. Allison, Benjamin D. Hopkins, Lewis C. Cantley, David S. Ludwig, Marcus D. Goncalves

**Affiliations:** 1 Department of Medicine, NYU Langone Health, New York, NY 10016, USA; 2 Department of Medicine, Weill Cornell Medicine, New York, NY 10065, USA; 3 Department of Population Health Sciences, Weill Cornell Medicine, New York, NY 10065, USA; 4 Cold Spring Harbor Laboratory, Cold Spring Harbor, NY 11724, USA; 5 Northwell Health, Manhasset, NY 11030, USA; 6 Faculty of Applied Bioscience, Department of Nutritional Science and Food Safety, Tokyo University of Agriculture, Setagaya, Tokyo 156-8502, Japan; 7 The New Balance Foundation Obesity Prevention Center, Boston Children’s Hospital and Harvard Medical School, Boston, MA 02215, USA; 8 Steno Diabetes Center Copenhagen, 2730 Herlev, Denmark; 9 Department of Pediatrics and USDA Children’s Nutrition Research Center, Baylor College of Medicine, Houston, TX 77030, USA; 10 Department of Epidemiology and Biostatistics, Indiana University School of Public Health-Bloomington, Bloomington, IN 47405, USA; 11 Department of Physiology and Biophysics, Weill Cornell Medicine, New York, NY 10065, USA; 12 Department of Cell Biology and Dana-Farber Cancer Institute, Harvard Medical School, Boston, MA 02215, USA; 13 Lead contact

## Abstract

This study explored the effects of three diets differing in the amount and type of carbohydrates on peripheral immune markers. After a 15% weight loss with a very-low-carbohydrate diet (VLCD), participants were randomized to isocaloric diets: VLCD, high-carbohydrate diet with high starch content (HC-Starch), or high-carbohydrate diet with high sugar content (HC-Sugar). Changes in peripheral immune markers were assessed in 44 participants using multimodal analyses before randomization and after 10 weeks on the diet. In HC-Starch, cytometry by time of flight (CyTOF) revealed significant shifts in proportions of circulating natural killer (NK) cells and increased activation markers in NK cells, monocytes, T cells, dendritic cells, and neutrophils. In agreement, transcriptomics showed upregulation of immune and metabolic pathways with HC-Starch but reductions with HC-Sugar. Cytokine analysis and mouse experiments suggested that increased adipsin and complement activation could underlie these changes. Following weight loss with VLCD, starch-rich diets serve as an unrecognized driver of inflammation. This study was registered at ClinicalTrials.gov: NCT03394664.

## In brief

Dantas et al. found that in participants with obesity, a diet rich in refined grains increases immune activation in leukocytes compared to an equicaloric diet rich in sugar or a very-low-carbohydrate diet (VLCD) following a period of weight loss with VLCD.

## Graphical Abstract

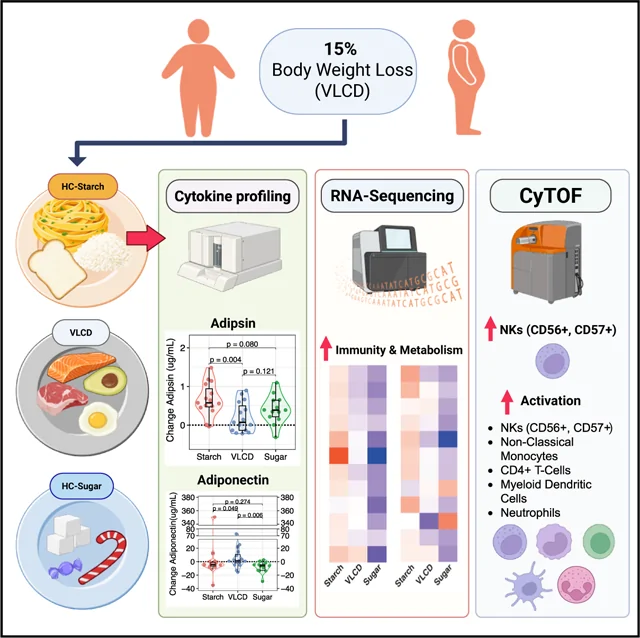

## INTRODUCTION

Dietary nutrients play a central role in shaping immune responses by modulating both cellular metabolism and systemic inflammatory tone. Modulating nutrient availability through dietary interventions can reshape immunity, alter chronic inflammation, and enhance cancer therapy.^1–9^ Obesity, a state of chronic nutrient excess, is associated with systemic low-grade chronic inflammation, which contributes to the increased risk for complications such as cardiovascular disease and cancer.^10–12^ Dietary interventions such as caloric restriction, time-restricted feeding, amino acid alternation, and very-low-carbohydrate diets (VLCDs) promote weight loss and exert profound effects on the immune system.^4,5,7,13^ Thus, the strategic manipulation of diet holds potential as a therapeutic tool to harness immune function.

Immune cells have specific metabolic demands based on their profile, differentiation, and activation state.^3^ For example, proliferating and activated T and B cells are highly glycolytic, while quiescent or memory cells rely more heavily on oxidative phosphorylation and fatty acid oxidation.^14–16^ Similarly, macrophages, depending on their activation state, exhibit distinct metabolic profiles. Proinflammatory M1 macrophages primarily utilize glycolysis, whereas anti-inflammatory M2 macrophages rely on oxidative phosphorylation and fatty acid oxidation.^17,18^ By contrast, neutrophils use the pentose phosphate pathway to fuel the production of reactive oxygen species (ROSs) during the oxidative burst to fight pathogens.^19^ These metabolic dependencies render immune cells sensitive to nutrient fluctuations, positioning diet as a potential regulator of immune cell function and phenotype.^20^

Most calories consumed from the prevailing diets in agrarian societies are from carbohydrates. There has been a shift in the types of carbohydrates consumed over the past century, where the wide availability and low cost of refined grains and sugars facilitate broad consumption.^21,22^ Despite similar energy content, these carbohydrate types differ substantially in their absorption kinetics and metabolic effects. Refined grains (starch) have little fiber and are rapidly digested and absorbed compared to unprocessed grains, leading to rapid blood glucose spikes in the postprandial period, which can affect substrate partitioning, systemic metabolism, and, potentially, immunity.^23,24^ Previous studies have linked diets rich in these high-glycemic-index refined grains with an increase in systemic markers of inflammation.^25–29^ Consumption of added sugar, specifically those containing fructose, has also been shown in mouse models and human trials to be associated with chronic inflammation through several mechanisms, including altered hepatic metabolism, worsening insulin resistance, gut dysbiosis, and gut barrier dysfunction.^30–32^ However, the differential impact of these carbohydrates on the human immune system, and particularly on circulating leukocytes, remain understudied. Here, we examined the immunologic consequences of reintroducing refined carbohydrates after substantial weight loss induced by a VLCD. Using high-dimensional immune profiling, including multiplexed cytokine analysis, whole-blood transcriptomics, and cytometry by time of flight (CyTOF), we tested the hypothesis that reintroduction of refined starch and sugar would have divergent effects on peripheral immune activation.

## RESULTS

### Study design

Whole-blood and plasma samples were analyzed from 44, 42, and 40 participants of the FB4 study for cytokine, RNA sequencing (RNA-seq), and CyTOF analyses, respectively (Table S1). The mean age was 35 years, and BMI ranged from 25.3 to 31.0 kg/m^2^ at enrollment. As part of the study protocol, participants consumed a hypocaloric VLCD at home and lost an average of 15.25% (±2.20%) over 3–4 months.^33^ They were then admitted to a residential research center, and the diet prescription was altered to deliver sufficient calories for weight-loss maintenance, as previously described.^34^ All participants were in active ketosis at start (pre-randomization), as indicated by high levels (2.70–3.09 mM) of serum β-hydroxybutyrate (BHB; Table S2). Glucose, insulin, triglycerides, and C-reactive protein (CRP) levels were in the low-normal range for most participants, and there were no significant differences among participants who were subsequently randomized to different dietary intervention groups (Table S2).

After 3 weeks of residential isocaloric VLCD feeding, participants were randomized to continue a VLCD or to receive one of two high-carbohydrate diets, containing 20% of total energy from either starch in refined grains (HC-Starch) or added sugar (HC-Sugar). At 10 weeks post randomization (end), participants in the VLCD group remained in dietary ketosis, albeit with a modest reduction in the average BHB level (Figure 1B; Table S2). In both high-carbohydrate groups, the BHB level was completely suppressed (Figure 1B; Table S2). As expected, post-prandial glucose levels were significantly higher in the HC-Sugar and HC-Starch groups, as previously reported (Figure 1C).^34^ There were no significant changes in hemoglobin A1c (HbA1c), despite observing higher levels at endpoint in the starch and sucrose groups (Figures S2A and S2B). All three intervention groups showed reduced levels of CRP following randomization, with the most significant in those consuming HC-Sugar, followed by HC-Starch (Table S2). All groups achieved levels of CRP considered low (<10 mg/L) by most clinicians and researchers.

### Diets rich in starch or sugar differentially alter plasma adipsin and adiponectin levels

Because participants consumed a VLCD for more than 13 weeks prior to randomization, the start and end time points provided a unique setting to examine the effects of reintroducing refined carbohydrates on the immune system. We began our investigation by measuring the change in concentration of 54 cytokines and inflammatory mediators in plasma (Table S3). We observed significant changes in the blood levels of two adipokines, adipsin (also known as complement factor D) and adiponectin, across the different diet groups. Adipsin, which is mainly produced in the adipose tissue, increased the most in the HC-Starch group compared to the VLCD control group (Figure 1D; Figure S2C). In contrast, adiponectin significantly decreased in both the HC-Sugar and HC-Starch groups while remaining unchanged in the VLCD control group (Figure 1D; Figure S2D).

### Diets rich in starch or sugar drive distinct blood leukocyte transcriptional profiles

To assess the effects of refined carbohydrates on leukocytes, we performed RNA-seq of whole blood followed by gene set variation analysis (GSVA), a type of gene set enrichment analysis capable of scoring single samples.^35,36^ To compare differences in pathways, we subtracted start from end scores and compared differences among the diets. GSVA revealed significant changes to pathways related to immunity and metabolism with the HC-Starch and HC-Sugar groups exhibiting opposite trends in pathway activation. The HC-Starch group scored the highest in immune activation and metabolic pathways, whereas the HC-Sugar group scored the lowest in these pathways (Figures 2A and 2B; Figures S3A–S3C; Table S4). Specifically, HC-Starch exposure induced the activation of pathways related to innate and adaptive immunity such as bacteria and fungus defense, complement activation, antigen presentation, T cell activation, and cytokine secretion (Figure 2A; Figures S3A and S3B; Table S4). The immune activation was mirrored by an upregulation of anabolic metabolism pathways such as mTOR activation, carbohydrate metabolism, amino acid transport, and catabolism (Figure 2B). In agreement, the HC-Starch group also showed the highest scores in pathways related to mitochondria activity (Figure S3C). Conversely, the HC-Sugar group resulted in the lowest immune and metabolic activation scores for all three diets, suggesting broad immune pathway downregulation (Figures 2A and 2B).

Next, we assessed whether the high-carbohydrate diets induced changes in the expression of cell receptors related to the differentially expressed adipokines in the plasma. Adipsin participates in the activation of the alternative pathway of the complement system by cleaving complement factor B, leading to the activation of the alternative pathway C3-convertase (C3bBb). C3bBb cleaves C3 into C3b and C3a; the latter is a potent anaphylatoxin also capable of stimulating insulin production by activating its receptor, C3aR (gene: C3AR1).^37,38^ In agreement with the decreased immune activity observed in the pathway analysis, we found that HC-Sugar significantly decreased the expression of C3AR1 in leukocytes (Figure 2C). Similarly, the receptor for adiponectin (ADIPOR1) showed opposite expression trends, with upregulation in the HC-Starch group and downregulation in the HC-Sugar group (Figure 2D). Because all the immune populations in circulation are included when doing bulk RNA-seq of whole blood, it is not possible to identify which particular cell types drive these changes. However, because receptor expression is restricted to certain subsets, we can infer the most likely contributing populations. To do this, we analyzed a publicly available dataset of gene expression in circulating immune cells (absolute immune signal [ABIS])^39^ and found that C3AR1 is expressed most highly in basophils, followed by non-classical, intermediate, and classical monocytes, whereas ADIPOR1 is predominantly expressed in neutrophils (Figures S3D and S3E).

### CyTOF identifies immune cell activation signatures with starch-rich diets

The cytokine and gene expression changes observed following the reintroduction of refined carbohydrates may simply reflect a change in the number or activation state of different cell populations. Therefore, we evaluated the blood cell composition of whole blood over time using CyTOF (Figures S4A–S4D and S5). There were no changes in cell populations at start and end for those in the VLCD arm; however, we found that HC-Starch induced a significant increase in the proportion of early natural killer (NK) cells (C56^+^CD57^−^) and a reduction in the proportion of late NK cells (CD56^+^CD57^+^), which concomitantly showed higher levels of pNF-κB (Figures 3A–3C; Table S5). Given the importance of IL-15 in NK cells homeostasis and differentiation, we analyzed the expression of its receptor, IL-15RA, in the RNA-seq and found it significantly increased in the HC-Starch group (Figure S4E). Because NK cells are some of the most important producers of IFN-γ, we also analyzed their RNA expression and found that it was significantly increased in the HC-Sugar group (Figure S4F). Consistent with this, IFNG-AS1, a long noncoding RNA that positively regulates IFNG expression and IFN-γ production, was also increased in the HC-Sugar group (Figure S4G).

Circulating monocytes can be categorized into three distinct types: classical (CD14^+^, CD16low), non-classical (CD14^+^, CD16high), and an intermediary population known as transitional monocytes. While classical monocytes are believed to be critical for the inflammatory response because they can differentiate into tissue macrophages, non-classical monocytes are believed to be anti-inflammatory and maintain vascular homeostasis. Human leukocyte antigen class II (HLA-DR) is essential for antigen presentation and is correlated with monocyte function and activation. In the non-classical monocyte population, HLA-DR was upregulated by reintroducing carbohydrates in both the HC-Starch and HC-Sugar groups (Figure 3D). Furthermore, CD38, a marker of monocyte activation, was also increased in the HC-Starch compared to the control VLCD (Figure 3E). Similarly, in the RNA-seq dataset, CD86, another marker of activation, remained unchanged in the HC-Starch group, while its expression was downregulated in the VLCD and HC-Sugar (Figure S4H). Signs of immune activation were also evidenced by the rise of pNF-κB in total CD4^+^ T cells and myeloid dendritic cells (mDCs) of participants in the HC-Starch group (Figures 3F and 3G). Upon activation, T cells upregulate the checkpoint receptor PD-1. Unexpectedly, we observed higher PD-1 expression on both CD4 and CD8 T cells in the HC-Sugar group, but not in the HC-Starch group, despite the latter showing the highest levels of pro-inflammatory transcription factor pNF-κB (Figures S4I and S4J; Table S5).^40^ Neutrophils were also affected by carbohydrate exposure. While both the HC-Starch and HC-Sugar groups showed similar levels of p-STAT3, neutrophils from the HC-Starch group significantly increased pNF-κB (Figures 3H and 3I). To gain further insight into the mechanisms and mediators underlying inflammation in the HC-Starch group, we performed a correlation analysis integrating the different data modalities. In this group, changes in plasma adipsin levels correlated with changes in CXCL8 transcript abundance, a potent neutrophil chemoattractant, as well as with neutrophil p-STAT3 measured by CyTOF (Figures S6A and S6B). Changes in plasma adipsin also correlated with total monocyte and CD8^+^ T cell numbers and with p-STAT3 in CD8^+^ central memory T cells (Figures S6C–S6E). Finally, total NK cell number correlated with expression of the C3a receptor, C3AR1 (Figure S6F).

### Starch refeeding activates complement and circulating immune cells in mice

To investigate underlying mechanisms and assess causality, we next turned to a controlled mouse model of starch and sugar refeeding. In these experiments, we simulated starch consumption using maltodextrin, a water-soluble glucose polysaccharide derived from starch, and modeled sugar consumption with an equicaloric sucrose solution. Although both maltodextrin and sucrose have the same caloric density and digestibility, maltodextrin hydrolyzes exclusively into glucose, whereas sucrose hydrolyzes into one molecule each of glucose and fructose. Following a 15-h fast, mice were orally gavaged with 2 g/kg of either a maltodextrin or sucrose solution, and levels of glucose, insulin, and adipsin were assessed from serum at different time points (Figures S7A–S7C). As predicted, maltodextrin increased glucose and insulin levels significantly more than sucrose. Interestingly, adipsin followed the opposite trend, decreasing more significantly with maltodextrin than with sucrose (Figure S7C). We hypothesized that the decrease in adipsin following maltodextrin consumption resulted from the activation of the alternative pathway of complement. To test this hypothesis, we performed a western blot of the serum and probed it with antibodies to show C3’s degradation fragments, which would appear as a consequence of complement activation (Figure S7D). Using mouse plasma treated with cobra venom factor (CVF) to activate complement as a positive control, we observed increases in the formation of C3 aggregates (C3-X) at 40 min and increased levels of the degradation fragments C3c and C3d after 60 min in the maltodextrin group (Figure S7D). Furthermore, after 2 h from the oral gavage with maltodextrin, degradation fragments from C3 could be detected bound to the surface of CD45^+^ cells (Figure S7E). These data suggest complement activation following consumption of maltodextrin.

To assess the effect of maltodextrin on the immune system, we evaluated activation markers in T cells, neutrophils, and monocytes. In this analysis, we used water as a control because both sucrose and fructose can independently influence immune responses and, therefore, introduce a confounding effect.^31,32,41^ We found that, after an hour of the maltodextrin challenge, both CD4 and CD8^+^ T cells increased the expression of the early activation markers CD69 and CD25 (Figures S7F–S7I). After 2 h, NK cells increased the expression of the activation marker TIGIT, while neutrophils decreased the expression of CD62L, suggesting recent activation (Figures S7J and S7K). Maltodextrin also increased the proportion of Ly-6C^+^ monocytes (classical monocytes), which expressed higher levels of CD62L and CD11b, also suggesting immune activation with maltodextrin (Figures S7L–S7N). Overall, these experiments show that starch consumption leads to complement and immune activation.

## DISCUSSION

Dietary nutrients are known to have profound effects on physiology and metabolism, predisposing to several chronic diseases. Here, we examine the effects of carbohydrate reintroduction on the immune phenotype, including changes in circulating cytokine and adipokine levels, leukocyte composition, and transcriptomic profiles following randomization to diets enriched in refined starch or added sugar after VLCD feeding.

Collectively, our study reveals that a diet high in refined starch leads to broad immune activation when compared to a diet high in sugar or a continued VLCD. Specifically, HC-Starch increased circulating adipsin, activated transcriptional programs related to innate immunity and complement, shifted NK cell proportions, and induced activation markers across multiple leukocyte populations, while HC-Sugar showed an opposing pattern of immune pathway suppression. Refined grains have been linked to markers of low-grade, chronic inflammation, with one established mechanism being their ability to increase postprandial blood glucose levels, as quantified by the glycemic index (GI).^23–29^ Hyperglycemia has been shown to induce the secretion of pro-inflammatory cytokines, and increased pNF-κB resulted in monocyte activation.^42,43^ Furthermore, in a model of autoimmunity, hyperglycemia led to immune activation and disease progression by promotion of the Th17 T cell profile. Refined starch has a higher GI than sugar, a difference that may have been augmented by the run-in VLCD,^44^ which reduces β cell responsiveness to oral carbohydrate.^34^ Our data suggest that the exaggerated glycemic excursion with starch leads to activation of the alternative complement pathway. Complement activation results in the production of C3a, a potent anaphylatoxin,^37^ to which several circulating immune cells, such as neutrophils and monocytes, possess receptors. For example, neutrophils are important targets for C3a, which may explain why the HC-Starch group showed higher levels of pNF-κB in this cell population.

Postprandial hyperglycemia may engage complement through multiple routes. Elevated glucose promotes non-enzymatic glycation of cell surface proteins, generating neoepitopes recognized by mannose-binding lectin and ficolins that can activate the lectin complement pathway.^45,46^ Our mouse data further suggest that starch-derived glucose triggers alternative pathway activation, as evidenced by C3 degradation fragments and a concomitant decrease in circulating adipsin, consistent with its utilization during active complement amplification. Together, these parallel mechanisms may account for the broad immune activation observed with HC-Starch but not HC-Sugar, where the lower glycemic excursion and suppression of C3AR1 expression may limit complement-driven inflammation.

The complement activation observed with HC-Starch may have both metabolic and immunological consequences. In addition to its role as an anaphylatoxin, C3a also functions as an insulin secretagogue.^37^ Lo et al. showed that adipsin-generated C3a stimulates β-cell insulin secretion through C3aR and that adipsin-deficient mice develop glucose intolerance due to impaired insulin output.^37^ We speculate that this response may be beneficial for glycemic homeostasis in the context of high-GI diets. However, several circulating immune cells express C3AR1, and this pathway may account for the increased NF-κB levels observed in neutrophils, late NK cells, myeloid dendritic cells, and T cells in humans consuming HC-Starch.

While these acute effects of C3a on immune cells may explain the activation phenotype, the sustained elevation of plasma adipsin in the HC-Starch group raises the question of what drives its chronic increase. This effect was not accompanied by changes in BMI, suggesting that the rise in adipsin may reflect changes in adipocyte secretory function rather than adipose tissue expansion. Chronic engagement of the alternative pathway by repeated postprandial glucose excursions may have driven compensatory upregulation of adipsin production to sustain complement-mediated insulin secretion. Future mechanistic studies, including detailed body composition and adipose tissue analyses, will be needed to test this hypothesis.

In contrast to adipsin, which increased selectively in the HC-Starch group, adiponectin decreased in both high-carbohydrate groups while remaining unchanged with continued VLCD. Higher circulating levels of adiponectin are associated with increased HDL cholesterol, reduced insulin resistance, and a lower risk of cardiovascular disease and type 2 diabetes.^47^ Population studies also show that elevated adiponectin levels are linked to lower adiposity, more favorable lipid profiles, and reduced systemic inflammation.^48^ Circulating adiponectin levels are dependent not only on fat mass but also on the macronutrient composition of the diet, with several studies indicating that diets rich in carbohydrates and low in fat are associated with decreased circulating adiponectin concentrations.^49,50^ Our data are consistent with this pattern, as both HC-Starch and HC-Sugar reduced adiponectin relative to VLCD.

At the cellular level, the most significant changes in the proportion of circulating immune cells were observed in the NK cell compartment of the HC-Starch group. NK cells provide rapid and effective responses to infected or transformed cells, playing a crucial role in the body’s innate immune defense. Here, early NK cells (CD56^+^, CD57^−^) increased, whereas late NK cells (CD56^+^, CD57^+^) decreased. Early NK cells are believed to produce higher levels of cytokines, whereas late NK cells are terminally differentiated and have higher cytotoxic capability.^51,52^ Late NK cells also displayed higher levels of pNF-κB in the HC-Starch group, suggesting that the differences observed in NK circulating proportions could be related to the activation of late NK cells and their migration to tissues, which was not captured in this study.

Recently, a VLCD was shown to modulate immune-related pathways and induce changes in adaptive immune cell populations.^53^ Our data add to this work by defining the key cytokines, pathways, and cells that are altered in both the innate and adaptive immune system when refined carbohydrate-containing diets are reintroduced after VLCD. For example, our GSVA analysis suggests that starch exposure may increase T cell activation. This finding is supported by the rise in pNF-κB levels in mDC and CD4^+^ T cells in the HC-Starch group. Myeloid cell populations also appear to be activated with the reintroduction of carbohydrates, as suggested by the upregulation of the major histocompatibility complex (MHC) class II molecules and CD38 in non-classical monocytes. These results highlight diet-induced changes in immune cell phenotypes, activation markers, and cytokine-responsive pathways that are not captured by more traditional markers of inflammation, such as CRP, which lack sensitivity in states of low-grade inflammation.^54–56^

In addition to its effects on systemic glucose homeostasis, fructose may directly modulate immune cell metabolism and function.^31,57^ For example, consumption of fructose-containing sugars significantly decreases the capacity of neutrophils to engulf bacteria.^58^ Our data suggest that this response may be due to reduced C3AR1 expression, which blocks the pro-inflammatory effects of the alternative complement pathway. However, our findings contrast with several lines of evidence indicating pro-inflammatory effects of fructose.^30,59^ The discrepancy may relate to the dose of fructose (∼10% of energy intake), interacting dietary components (e.g., the relatively high overall fiber content), or the metabolic state of our participants after weight loss on a carbohydrate-restricted run-in diet. Importantly, the reduction in inflammatory pathways in the HC-Sugar group should not be interpreted as an overall benefit of sucrose or fructose consumption, as suppression of complement-mediated immune responses could impair host defense. Although the 10-weeks test-diet period is relatively long compared to most published feeding studies, the findings may not reflect the chronic effects of these diets, due to the possibility of ongoing physiological adaption to a major change in nutrients.^34,60,61^ Ongoing work aims to delineate the direct versus indirect effects of fructose on immune function, particularly through detailed investigations into its impact on C3AR1 expression and complement pathway activation.

### Limitations of the study

Our study was limited by the focus on circulating immune cells and not those located in tissues. This constraint prevented us from further exploration of the differences in NK cell proportions and whether these or other cells were enriched in tissues or suppressed from leaving the bone marrow as suggested by several recent papers.^2,62,63^ Similarly, changes in plasma cytokines and chemokines do not provide accurate information about systemic immune cell trafficking or changes in tissue-resident immune cells. Recent studies have also shown that VLCDs and vegan diets dramatically change the microbiome, which could be responsible for changes in immunity.^53,63^ However, we did not analyze whether the changes in the immune phenotype respond to changes in gut microbiota. Lastly, we were limited by the lack of immune functional assays because the blood samples were immediately fixed at the time of collection. In the absence of functional assays, it is not possible to conclude whether the changes induced by either diet are associated with impaired or enhanced immune function. Similarly, because bulk RNA-seq of whole blood encompasses all circulating leukocytes, changes in pathways and genes cannot be attributed to a particular population. Despite these limitations, this study is the first to thoroughly assess changes in the immune phenotype that occur after reintroducing dietary refined grains and sugar after a prolonged period of abstinence. Our results highlight the importance of dietary nutrients for immune function and complex interactions among diet, metabolism, and the immune system.

### RESOURCE AVAILABILITY

#### Lead contact

Further information or other requests should be directed to and will be fulfilled by the lead contact, Dr. Marcus Goncalves (marcus.goncalves@nyulangone.org).

#### Materials availability

No unique reagents or tools were generated for this study.

#### Data and code availability

- All data necessary to support the conclusions of this study are provided in the paper and its supplemental information.
- No new code was generated for this study.
- Bulk RNA-seq data were deposited in the GEO: GSE278745.
- Any additional information required to reanalyze the data reported in this paper is available from the lead contact upon request.

## STAR★METHODS

### EXPERIMENTAL MODEL AND STUDY PARTICIPANT DETAILS

#### Experimental design

This study presents exploratory analyses from the Framingham, Boston, Bloomington, Birmingham, and Baylor study (FB4) (ClinicalTrials.Gov, NCT03394664). The full details of the study have been previously reported.^72^ Briefly, FB4 was a randomized controlled feeding trial designed to assess the impacts of dietary carbohydrate and sugar on body fat and metabolism, independent of caloric content. Eligible study participants were males and females aged 18–50 years, with a BMI ≥27 kg/m^2^, with no cardiovascular disease or diabetes. Specimens for this ancillary study were collected for cohorts 3 and 4 of the FB4 trial (*n* = 54) (Figure S1). The trial included run-in and residential phases (Figure 1A). During the run-in phase, participants received weekly home delivery of VLCDs prepared by Metabolic Meals (St. Louis, MO). This diet (7.5% of energy from carbohydrate without refined grains or added sugars, 67.5% from fat, and 25% from protein) was portioned to contain 40% caloric restriction based on each participant’s estimated energy needs. After achieving ∼15% weight loss, participants moved to a conference center for the residential phase. During the first 3 weeks of the residential phase, they received a VLCD featuring 100% of estimated energy needs for weight-loss maintenance. Participants were then randomized to an isocaloric VLCD (5% of energy from carbohydrate, 77% fat, and 18% protein) or one of two carbohydrate-rich diets. Both carbohydrate-rich diets had the same macronutrient composition (57% carbohydrate, 25% fat, and 18% protein), with 25% of calories derived from whole grains. However, the diets differed in that 20% of total energy came either from starch in refined grains (HC-Starch) or added sugar (HC-Sugar). Further dietary information and menu samples have been previously reported.^72^ Blood was collected after a 12-h overnight fast prior to randomization (START of trial) and again at the END of a 10-weeks test diet period after randomization. The primary outcome for the FB4 trial was body fat mass. In this ancillary study, we compared START and END blood samples, using high-dimensional single-cell mass cytometry (CyTOF), multianalyte cytokine profiling, and RNA-sequencing to explore changes in the immune system with the diets.

#### Mouse experiments

C57BL/6 male mice, aged 12–16 weeks were used for all experiments. The mice were kept under a 12-h light/dark cycle at a temperature of 22°C with unrestricted access to rodent chow (PicoLab Rodent 20, 5053, Lab Diet) and water. For the starch tolerance tests, mice were fasted for 5 h the morning of the experiment and then were oral gavaged with 2g/kg of a sucrose or maltodextrin solution (200mg/mL in water). Glucose was monitored using a glucometer (OneTouch Ultra 2) and blood was collected using either serum (Microvette 16.440.100) or plasma capillary tubes (Microvette #CT-CB300-K2EDTA). For the flow cytometry experiments in whole blood, mice were fasted overnight for 15 h in environmental control cabinets (CAB-16, Sable Systems International) at a temperature of 26.5°C as previously described.^73,74^ On the day of the experiment mice were gavaged with a maltodextrin solution (200mg/mL, 2g/Kg). Mice were then humanely euthanized one or two hours later and their blood collected by cardiac puncture in K3-EDTA tubes (Microvette #MV-E-500). Gating strategy for mouse experiments is shown in Figure S8.

#### Ethics

The institutional review board at Boston Children’s Hospital approved the study, and written informed consent was obtained from all participants. The study was registered in clinicaltrials.gov: NCT03394664, and participants used in this ancillary study were recruited from May 2019 to October 2019. All mouse experiments were reviewed and approved by IACUC under mouse protocol: PROTO202400023 at NYU Langone Health.

### METHOD DETAILS

#### Blood collection and preservation

Whole blood samples for CyTOF were collected in lithium-heparin tubes (BD vacutainer BD- 367874) and immediately aliquoted into 15 mL conical tubes and fixed using SmartTube Proteomic Stabilizer (cat no. PROT1), per the manufacturer’s instructions. Blood was preserved at −80°C until the day of the analysis. Whole blood for RNA-Sequencing analysis was drawn directly to PAXgene tubes (cat. No.762165) following the manufacturer’s instructions.

#### Cytokines

A multiplexed analysis of circulating cytokines was performed by Eve Technologies (Calgary, Canada) performed using the 48-plex Discovery Assay. This panel offers a comprehensive coverage of cytokines covering extensively studied pro-inflammatory markers such as IL-6 and TNF-α to anti-inflammatory cytokines such as IL-10. It also includes several chemokines and growth factors that, in combination with the CyTOF data could provide a comprehensive picture of immune cell trafficking. Since one of the study’s endpoints was changes in body composition, including adipose tissue, we added a 5-plex Adipokine Discovery Panel (Eve Technologies) to assess the effects of carbohydrates on adipose tissue. We additionally measured leptin in a separate aliquot using a custom-made Luminex panel (R&D Systems) and a L-200 Luminex machine (Biorad). Adipsin and insulin levels in mice were measured using a luminex kit from Millipore and an ELISA from Crystal Chem (#90080). Due to sample integrity issues and out of range values, data from one participant were excluded; however, all other processed samples were included in the analysis.

#### Whole blood RNA-sequencing

Total RNA was extracted from fixed whole blood using Qiagen PAXgene Blood RNA Kit following the manufacturer’s instructions (Qiagen, Hilden, Germany). RNA quality control was performed using Qubit 2.0 Fluorometer (Life Technologies, Carlsbad, CA, USA) and RNA integrity was checked with 4200 TapeStation (Agilent Technologies, Palo Alto, CA, USA).

Samples were initially treated with TURBO DNase (Thermo Fisher Scientific, Waltham, MA, USA) to remove DNA contaminants. rRNA and globin depletion was done using QIAseq FastSelect-rRNA HMR and -Globin kit (Qiagen, Germantown, MD, USA), per the manufacturer’s instructions. RNA sequencing libraries were constructed using the NEBNext Ultra II RNA Library Preparation Kit for Illumina following the manufacturer’s recommendations. Briefly, enriched RNAs were fragmented for 15 min at 94°C. First-strand and second-strand cDNA were subsequently synthesized and fragments were end-repaired and adenylated at 3^′^ends. Universal adapters were ligated to cDNA fragments, followed by index addition and library enrichment with limited cycle PCR. Sequencing libraries were validated using the Agilent Tapestation 4200 (Agilent Technologies, Palo Alto, CA, USA), and quantified using Qubit 2.0 Fluorometer (ThermoFisher Scientific, Waltham, MA, USA), as well as by quantitative PCR (KAPA Biosystems, Wilmington, MA, USA). The sequencing libraries were multiplexed and clustered on six flowcell lanes. After clustering, the flowcell was loaded on the Illumina instrument according to the manufacturer’s instructions. The samples were sequenced using a 2 × 150 Pair-End configuration. Initial bioinformatics analysis was done using HiSeq Control Software. Illumina HiSeq’s raw sequence files were converted into FASTQ files and de-multiplexed using Illumina’s bcl2fastq 2.17 software. For each index sequence identification, one mismatch was allowed.

#### RNA-sequencing analysis

Read alignments were done using Galaxy (https://usegalaxy.org/), where paired-end FASTQ files were processed with Cutadapt for adapter removal, and mapped using STAR (2-pass mapping) with the GENCODE v43 basic annotation was performed on genome assembly GRCh38 (hg38). Counts were obtained with featureCounts, and quality control was performed with FastQC and MultiQC. Data from 2 participants were excluded due to one of the timepoints failing quality control. Raw counts were normalized using DE-Seq2 (v.1.42.1) in R Studio (2023–10-31), with R (4.3.2), and exported using the function “counts”. Ensembl IDs were converted to HGNC gene symbols using the R package biomaRt (2.61.3). GSVA analysis was done with the GSVA R package (1.53.23) using normalized counts exported from DESeq2 and formatted using the function gsvaParam with default settings. GSVA analysis was ran using the gsva algorithm with default parameters. Gene Ontology gene sets (Biological Process) were retrieved using the gmtPathways function from the fgsea package (1.31.0). After GSVA analysis, we selected and metabolism related pathways and compared the effects of the diets by subtracting the START scores from END and these values were used to plot the heatmaps in Figure 2; Figure S1. Then, the resulting differences were compared among all diets using Kruskal-Wallis followed by Wilcoxon test and adjusted for multiple comparisons using the Benjamini and Hochberg (BH) method (Table S4).^75^

#### CyTOF

The protocol for using Fluidigm’s Maxpar Direct Immune Profiling Assay (MDIPA) tubes (cat. no 201325) in fixed whole blood using SmartTube was adapted from Rahman et al.^76^ The protocol for intracellular markers was adapted from previously published works.^77,78^ Samples from each dietary arm were randomly allotted, and START and END timepoints for each case were stained and run on the same day to minimize batch effect. Samples from 40 participants were stained with the MDIPA tubes, 36 were also stained with CD95, PD1, TIGIT, pSTAT3 (Tyr705), pNF-KB (p65, S529), p-S6 (S235/S236), which are referred as the “CyTOF (Other)” panel in Table S1. On the day of the analysis, the SmartTube stabilized frozen blood was thawed, and the red blood cells lysed using Thaw-Lyse solution (cat. no. 501351696) from SmartTube, per the manufacturer’s instructions. After thawing and lysis, samples were washed twice with Fluidigm’s Maxpar staining buffer and resuspended in 40 μl containg 0.1 KU/mL of sodium heparin (Sigma Aldrich #H3149–10KU) and 5 μl of Human TruStain FcX (Biolegend, 422302) or immunoglobulin receptor blockade. After 10 min, previously tittered antibodies against surface markers from Fluidigm CD95 (cat. No. 3162038B), PD1 (cat. No. 3165042B), and TIGIT (cat. No. 3209013B) were added and incubated for 15 more minutes. The samples were then diluted to the final volume of 270 ul with staining buffer containing 0.1 KU/ul of heparin and transferred to an MDIPA tube for 30 min at room temperature (RT). After two washes with 3mL of staining buffer, samples were fixed using a 1.6% PFA solution (Pierce, 28906) diluted in Maxpar PBS (Fluidigm S00125) for 10 min at RT. After a wash with 2mL of PBS, cells were delicately resuspended in 600 μl of cold methanol (−20C) to permeabilize the membrane and be stored at −80C. The next day, intracellular staining for pSTAT3 (Tyr 705), pNF-kB (p65, S529) and pS6 (S235/S236) was performed. Briefly, samples were retrieved from the −80 freezer and washed twice with staining buffer. The pellet was resuspended in 20 μl of a staining buffer solution containing 0.1 KU/mL units of heparin and previously titrated amounts of pSTAT3, pNF-kB and pS6 antibodies were added. After 30 min, cells were washed with 3mL of staining buffer and resuspended in 1mL of cell intercalating solution (Cell-ID Intercalator-Ir cat.no diluted 1:1,000 in Maxpar Fix and Perm Buffer (cat. No. 201067). After 30 min, cells were washed once with staining buffer and then with acquisition buffer. Lastly, samples were resuspended in 1 mL of acquisition buffer and filtered using the 70 μm filter available with Falcon’s flow cytometry tubes (cat. No. 352235). Before acquisition in a Helios system at Weill Cornell Englander Institute for Precision Medicine, samples were spiked with Ce140 EQ beads (cat. No. 201078). pSTAT3 (CST# 4113) and pNF-KB (BD #558393) antibodies were conjugated in-house with 169Tm and 159Tb using Fluidigm Maxpar ×8 Antibody labeling kits respectively, per the manufacturer’s instructions.

#### CyTOF analysis

Acquisition CyTOF files were normalized using Fluidigm’s pathsetter software. Sample cleanup and analysis was done in Flowjo following Fluidigm’s published technical note “Approach to Bivariate Analysis of Data Acquired Using the Maxpar Direct Immune Profiling Assay”, (PN 400248 B1). Gating strategy can be found in Figure S4. Data from 1 participant was removed from the final analysis as one of its samples failed quality control, and data from another participant were never stained due to incomplete red blood cell lysis. Samples from another two participants were never processed. As published by the Rahman group, whole blood fixated with SmartTube decreases the detection of some surface markers, especially of chemokine receptors.^76^ As reported in that manuscript, the following surface markers were omitted from the gating strategy (CD127, CCR7, CXCR5, CXCR3, CD25, CCR4, CCR6).^76^ The T-SNE plots were made using the R package CATALYST with previous batch correction with the package CyCombine. Then files were analyzed using FlowJo (v.10.10), gating strategy was derived from Fluidigm’s technical note “Approach to Bivariate Analysis of Data Acquired Using the Maxpar Direct Immune Profiling Assay”, excluding the markers mentioned above. Antigen expression was evaluated as median metal intensity (MMI) on arcsine transformed data. Differential analysis for changes in immune populations was done using the Kruskal -Wallis test and Wilcoxon’s test in the rstatix R package.

#### Plasma metabolites

The concentration of CRP was determined at the Clinical and Epidemiological Research Laboratory (CERLab) at Boston Children’s Hospital with an immunoturbidimetric assay on the Roche Cobas 6000 system (Roche Diagnostics - Indianapolis, IN), using reagents and calibrators from Roche. Fasting insulin levels were measured using ELISA (Crystal Chem #90115). Beta-hydroxybutyrate and triglycerides were measured by LabCorp using previously described methodology.^79,80^ Postprandial glucose levels (120 min after lunch) were obtained from previously published work.^34^

#### Flow cytometry

For each staining, 50 μl of mouse whole blood was pre-treated with TruStain FcX (Biolegend #101319) and then incubated with a panel of antibodies prepared in Brilliant Stain Buffer (BD #563794) for 20 min at room temperature. Red blood cell lysis was performed with distilled water as previously described.^74^ Samples were then acquired without fixation in a SONY ID7000 Spectral Cell Analyzer. Unmixing was performed in the ID7000 software and analysis in FlowJo (v.10.10).

#### Western Blot

Plasma samples were diluted 1:75 and denatured at 70°C for 20 min and ran onto 4–12% NuPAGE Bis-Tris ran in non-reducing conditions (Thermo Fisher # WG1402BOX). Then samples were transferred to PVDF membranes (0.22um) overnight at 10 volts, for 16 h in wet transfer cells (Bio-Rad Laboratories) and later blocked for one hour in SuperBlock (Thermo Fisher # 37515). Membranes were then blotted with the M213 (Complement Technology, 1:500) and anti-C3d (R&D, #AF2655-SP, 1:500) antibodies overnight. Membranes were then washed and revealed using an anti-goat HRP-linked antibody (Thermo Fisher #A15999, 1:2000) and imaged in a Chemidoc (Bio-Rad).

### QUANTIFICATION AND STATISTICAL ANALYSIS

All statistical analyses were performed in R (4.3.2) using R Studio (2023–10-31). Summary statistics presented in Table S1 were performed using the function tbl_summary of the R package gtsummary (2.0.2) with default settings. The effects of diets presented on the analysis of the cytokine, RNA-Seq and CyTOF data sets were evaluated by subtracting the values from the START timepoint to the END and then comparing these differences using Kruskal-Wallis test followed by Wilcoxon rank-sum test. Paired analysis in Tables S3–S5 were done with Wilcoxon signed-rank test with *p*p-values adjusted for multiple comparisons using Bonferroni’s adjustment. The statistical analysis plan was discussed and reviewed by two independent biostatisticians (Yushu Shi and Oleksandr Savenkov). Cases excluded for technical reasons are detailed in the section for each method, and all readings for those samples were removed prior to analysis.

### ADDITIONAL RESOURCES

The registry for the parent study can be found on ClinicalTrials.gov (NCT03394664): https://clinicaltrials.gov/study/NCT03394664. Bulk RNA Sequencing data were deposited in the Gene Expression Omnibus (GSE278745).

## Supplementary Material

1

Supplemental information can be found online at https://doi.org/10.1016/j.cpblue.2026.100122.

## Figures and Tables

**Figure 1.**
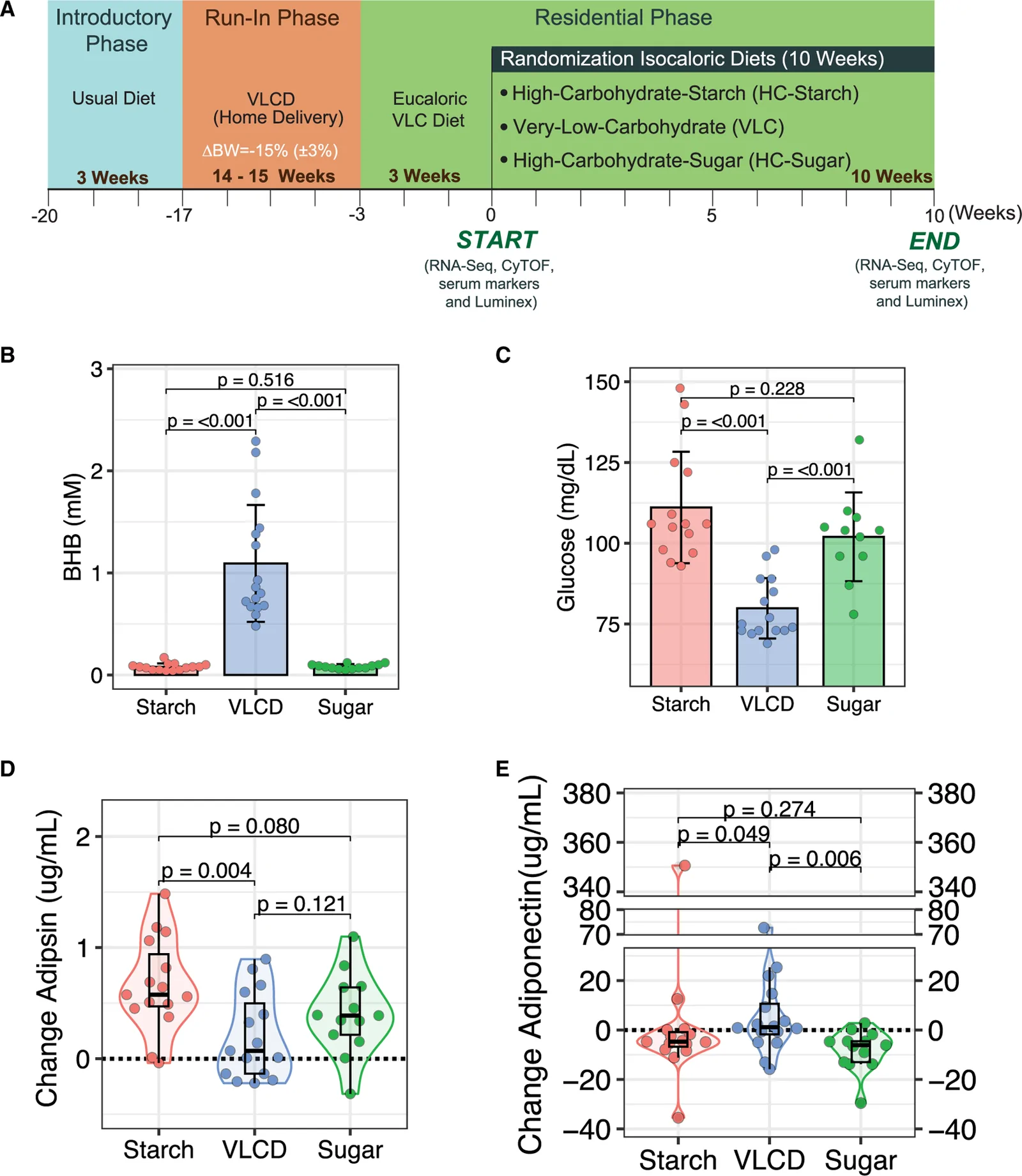
Study design and changes in circulating mediators (A) Experimental design describing the three phases of the study. (B–E) Fasting levels of BHB at endpoint (B), postprandial (2 h after lunch) glucose levels at endpoint (C), adipsin (D), and adiponectin (E). Comparisons in (B and C) were made using Wilcoxon’s test. Comparisons in (D and E) were made subtracting the start from the end values (change) followed by Kruskal-Wallis and Wilcoxon’s test. For box plots in (D) and (E), horizontal bars within boxes represent medians. Tops and bottoms of boxes represent 25th and 75th percentiles, and vertical lines extend to the 1.5× interquartile range. Error bars represent standard deviation (SD). Statistical significance was defined as *p* < 0.05.

**Figure 2.**
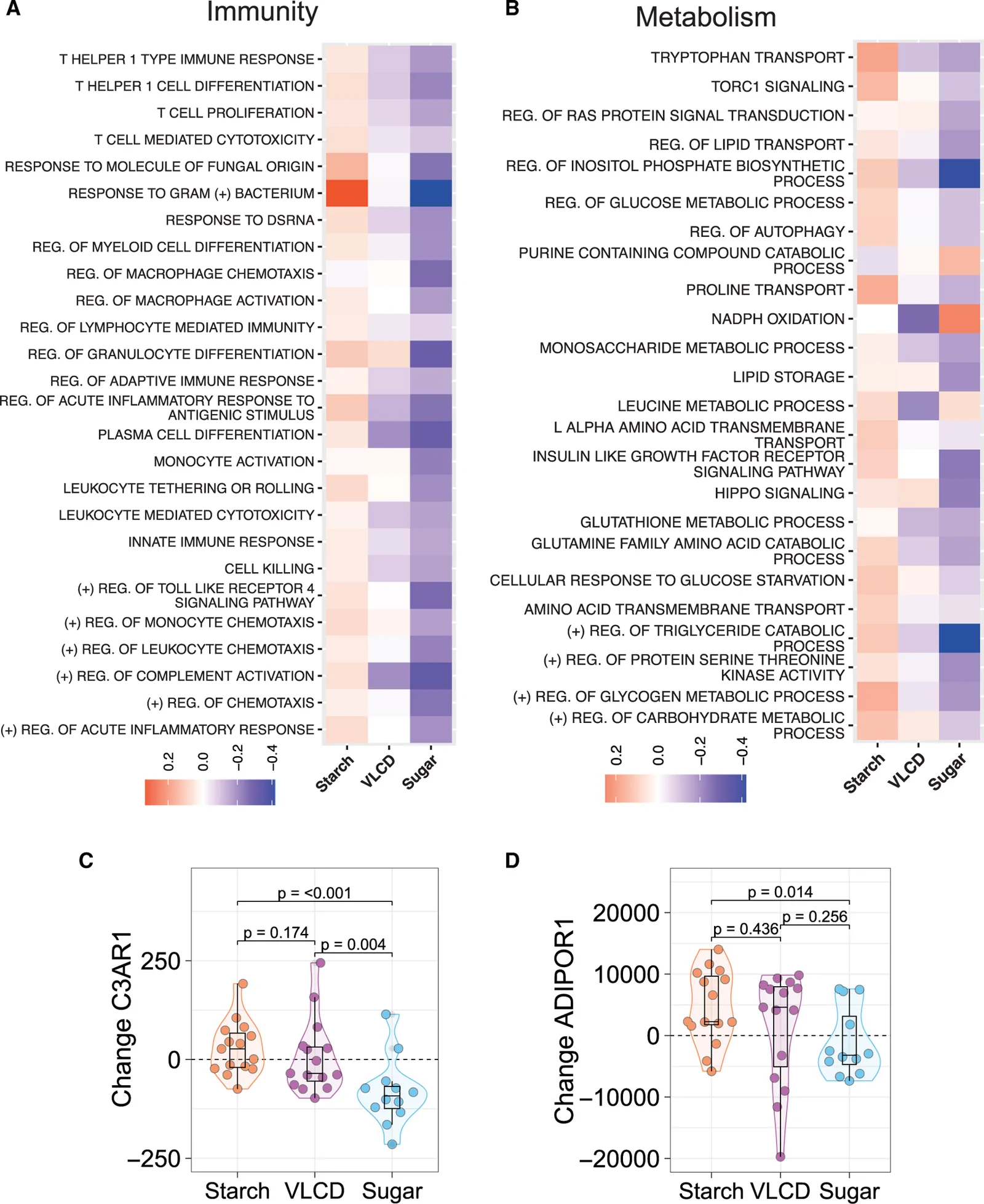
Diets with different carbohydrate composition after VLCD change the transcriptional signature of circulating leukocytes (A and B) Heatmaps showing changes between end and start time points for the GSVA scoring of pathways related to immunity (A) and metabolism (B) for the different diets. (C and D) Diet comparison of the differences in gene expression between end and start for the cytokine receptors C3AR1 (C) and ADIPOR1 (D). Heatmaps in (A) and (B) were made using the mean value for the change (end - start) of the GSVA pathways scores. Comparisons in (C) and (D) were made by subtracting the start from the end normalized counts (change) followed by Kruskal-Wallis and Wilcoxon’s test. For box plots in (C) and (D) horizontal bars within boxes represent medians. Tops and bottoms of boxes represent 25th and 75th percentiles, and vertical lines extend to the 1.5× interquartile range. Error bars represent standard deviation (SD). Statistical significance was defined as *p* < 0.05.

**Figure 3.**
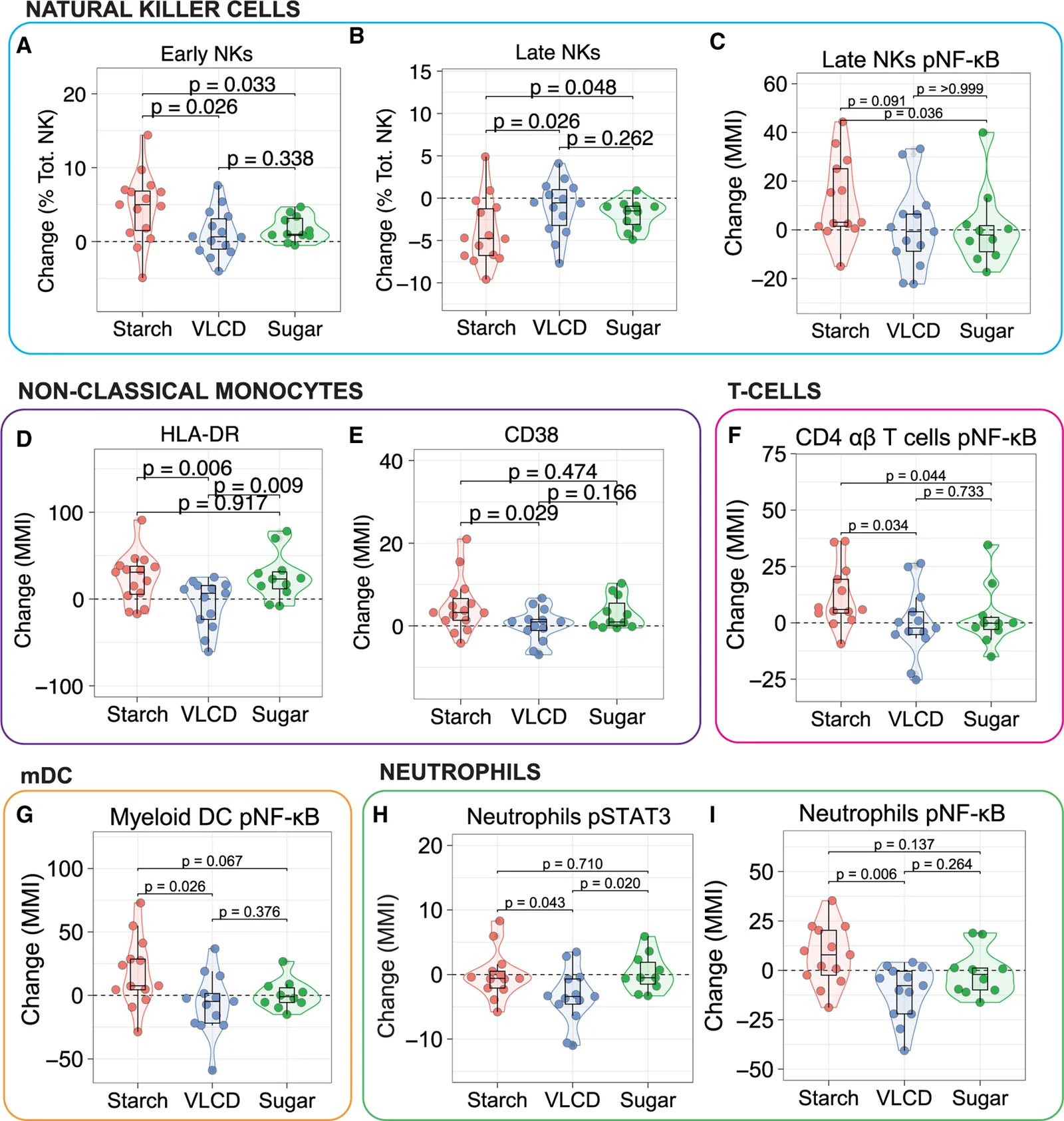
Changes in immune profiles following randomization to diets with different carbohydrate composition after VLCD (A and B) Comparison of changes in natural killer (NK) cell composition among the diets. (C) Comparison of changes in pNF-κB expression in the late NK cell subtype among the diets. (D and E) Comparison of changes in HLA-DR (D) and CD38 (E) expression in circulating non-classical monocytes with the different diets. (F) Comparison of changes in pNF-κB expression in total CD4αβ T cells. (G) Comparisons of changes in pNF-κB expression in myeloid DCs. (H and I) Comparison of changes in pSTAT3 (H) and pNF-κB (I) expression in neutrophils among the diets. Comparisons in (A–F) and (I) were made by subtracting the start from the end values followed by Kruskal-Wallis Wilcoxon’s test. For box plots, horizontal bars within boxes represent medians. Tops and bottoms of boxes represent 25th and 75th percentiles, and vertical lines extend to the 1.5× interquartile range. Error bars represent standard deviation (SD). Statistical significance was defined as *p* < 0.05.

**Table:** KEY RESOURCES TABLE

REAGENT or RESOURCE	SOURCE	IDENTIFIER

Antibodies		

Maxpar Direct Immune Profiling Assay (MDIPA)	Fluidigm	Cat# 201325
Human TruStain FcX	Biolegend	RRID: AB_2818986; Cat# 422302
CD95	Fluidigm	RRID: AB_3110292; Cat# 3162038B
PD1	Fluidigm	RRID: AB_2905648; Cat# 3165042B
TIGIT	Fluidigm	RRID: AB_2905649; Cat# 3209013B
Phospho-Stat3 (Tyr705) (M9C6)	CST	RRID: AB_2198588; Cat# 4113
Phospho-S6 (S235/S236) (N7-548)-175Lu	Standard BioTools	RRID: AB_2864737; Cat# 3175031D
Phospho-NF-kB (p65) (S529)	BD Biosciences	RRID: AB_647284; Cat# 558393
CD45RA	Biolegend	RRID: AB_10965547; Cat# 304130
CD4	Thermo Fisher	RRID: AB_10609337; Cat# 50-0041-82
CD8a	Biolegend	RRID: AB_2562100; Cat# 100748
CD3	Thermo Fisher	RRID: AB_469889; Cat# 53-0031-82
CD69	Biolegend	RRID: AB_493564; Cat# 104512
anti-Goat	Thermo Fisher	RRID: AB_2556668; Cat# SA5 10088
anti-C3	ComplementTech	Cat# M213
CD44	Biolegend	RRID: AB_2562451; Cat# 103047
CD62L	Biolegend	RRID: AB_2561537; Cat# 104441
CD25	BD Biosciences	RRID: AB_2722574; Cat# 564022
Ly-6C	BD Biosciences	RRID: AB_2737748; Cat# 562727
MHC II	BD Biosciences	RRID: AB_2738191; Cat# 563414
CD11b	Thermo Fisher	RRID: AB_2734870; Cat# 12-0112-83
Ly-6G	BD Biosciences	RRID: AB_1727562; Cat# 560601
CD45	Thermo Fisher	RRID: AB_469392; Cat# 17-0451-82
CD11c	Biolegend	RRID: AB_11204262; Cat# 117333
NKp46	Miltenyi	RRID: AB_2657612; Cat# 130-112-361
TIGIT	Thermo Fisher	RRID: AB_11149128; Cat# 46-9501-80
CD11b	Thermo Fisher	RRID: AB_2734870; Cat# 12-0112-83
CD27	Thermo Fisher	RRID: AB_1724035; Cat# 25-0271-82
CD161	Biolegend	RRID:AB_2562273; Cat# 108739
eFluor 506 Fix Viability	Thermo Fisher	Cat# 65-0866-14
Live/Dead Fix Near IR (775)	Thermo Fisher	Cat# L34975

Chemicals, peptides, and recombinant proteins		

Sodium Heparin	Sigma Aldrich	Cat# H3149-10KU
Pierce 16% Formaldehyde (w/v), Methanol-free	Thermo Fisher	Cat# 28906
Maxpar PBS	Fluidigm	Cat# S00125
Maxpar Cell Staining Buffer	Fluidigm	Cat# 201068
Maxpar Fix and Perm Buffer	Fluidigm	Cat# 201067
Maxpar ×8 Antibody Labeling Kit, 169Tm	Fluidigm	Cat# 201169A
Maxpar ×8 Antibody Labeling Kit, 159Tm	Fluidigm	Cat# 201159A
EQ Four Element Calibration Beads	Fluidigm	Cat# 201078
Cell-ID Intercalator-Ir 125 mM	Fluidigm	Cat# 201192A

Critical commercial assays		

Qiagen PAXgene Blood RNA Kit	Qiagen	Cat# 762164
TURBO DNase	Thermo Fisher	Cat# AM2238
QIAseq FastSelect -rRNA/Globin Kit	Qiagen	Cat# 335376
NEBNext Ultra II RNA Library Prep Kit for Illumina	New England Biolabs	Cat# NEB#E7490

Deposited data		

Gene Expression Omnibus	GEO: GSE278745	https://www.ncbi.nlm.nih.gov/geo/query/acc.cgi?acc=GSE278745

Software and algorithms		

Galaxy	N/A	https://usegalaxy.org
Cutadapt	Marcel Martin: https://doi.org/10.14806/ej.17.1.200	https://cutadapt.readthedocs.io/en/stable/
FastQC	“FastQC”^64^	https://github.com/s-andrews/FastQC
MultiQc	Ewels et al.^65^	http://multiqc.info
featureCounts	Liao et al.^66^	https://github.com/galaxyproject/tools-iuc/blob/main/tools/featurecounts/featurecounts.xml
RStudio (2023-10-31)	N/A	https://posit.co/
STAR	Dobin et al.^67^	N/A
R (4.3.2)	N/A	https://www.r-project.org/
GENCODE v43 basic annotation	N/A	https://www.gencodegenes.org/human/release_43.html
DESeq2 (v.1.42.1)	Love M. et al.^68^	https://github.com/thelovelab/DESeq2
GSVA (1.50.1)	Hanzelmann S. et al.^35^	https://github.com/rcastelo/GSVA
FlowJoV10.6	BD Biosciences	https://www.flowjo.com/solutions/flowjo/downloads
CYTOF Software V6.0	Fluidigm	https://www.fluidigm.com/products-services/software
CyCombine	Pedersen CB et al.^69^	https://github.com/biosurf/cyCombine/.
CATALYST	Crowell H.L. et al.^70^	https://github.com/HelenaLC/CATALYST
ABsolute Immune Signal (ABIS) deconvolution	Monaco G et al.^39^	https://github.com/giannimonaco/ABIS
Gene Ontology gene sets	N/A	https://www.gsea-msigdb.org/
rstatix	Kassambara A.	https://github.com/kassambara/rstatix
ggvenn (0.1.10)	Linlin Y.	https://github.com/yanlinlin82/ggvenn
ggplot2	Wickham H.^71^	https://github.com/tidyverse/ggplot2

Other		

PAXgene tubes	BD Biosciences	Cat# 762165
SmartTube Proteomic Stabilizer	SmartTube	Cat# PROT1
Thaw-Lyse	SmartTube	Cat# 501351696
Maxpar ×8 Antibody Labeling Kit,165Ho	Fluidigm	Cat# 201165A
Maxpar X8AntibodyLabelingKit, 159Tb	Fluidigm	Cat# 201159A
Falcon 5mLRound Bottom Polystyrene Tube with CellStrainer Snap Cap 35mm	Corning Inc.	Cat# 353263
48-plex Discovery Assay	Eve Technologies	Cat# Human Cytokine 48-Plex Discovery Assay
5-plex Adipokine Discovery	Eve Technologies	Cat#Human Adipokine 5-Plex Discovery Assay
Leptin Luminex	R&D	Cat# LUBM398

## Highlights

- Refined grains are associated with increased levels of the complement factor adipsin
- Refined grains activate immune and metabolic pathways in circulating leukocytes
- Refined grains activate NKs, monocytes, CD4^+^ T cells, and neutrophils
- Mouse data support that complement is a mediator of starch-induced inflammation
