# Supplementary material for: Effects of refined carbohydrates on immune markers after weight loss on a very-low-carbohydrate diet: A randomized controlled feeding trial
Source: Cell Press Blue. Author manuscript; Available in PMC 2026 Oct 20. (PMC13679806; doi:10.1016/j.cpblue.2026.100122)
Supplement: Supplementary file 1 [file NIHMS2211996-supplement-1.pdf]

CPBLUE, Volume *1*

Supplemental information

Effects of refined carbohydrates on immune markers after weight loss on a very-low-carbohydrate diet: A randomized controlled feeding trial

Ezequiel Dantas, Yushu Shi, Jeshua Kim, Anirudh Murthy, Alexander Emmanuelli, Shiri Li, Tiffany Perrier, Tanvir Ahmed, Isaac Nathoo, Carolina E. Echeverria, Yingzhao Wang, Omar Abdallah, Tobias Janowitz, Maurice A. Hurd, Noriko Suzuki-Kemuriyama, Cara B. Ebbeling, David B. Allison, Benjamin D. Hopkins, Lewis C. Cantley, David S. Ludwig, and Marcus D. Goncalves

Figure S1

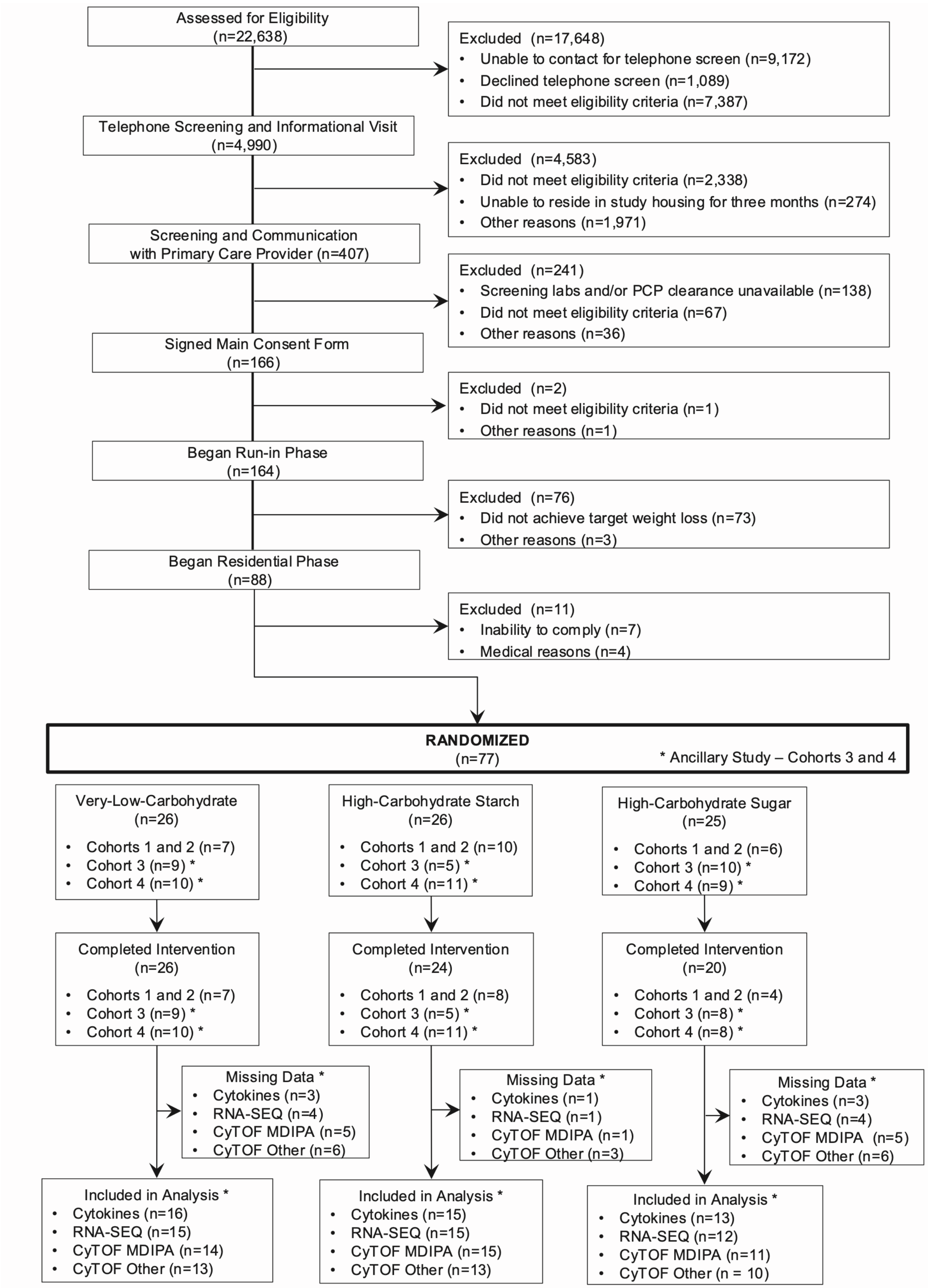

Supplementary Figure 1. CONSORT flow diagram.

Figure S2

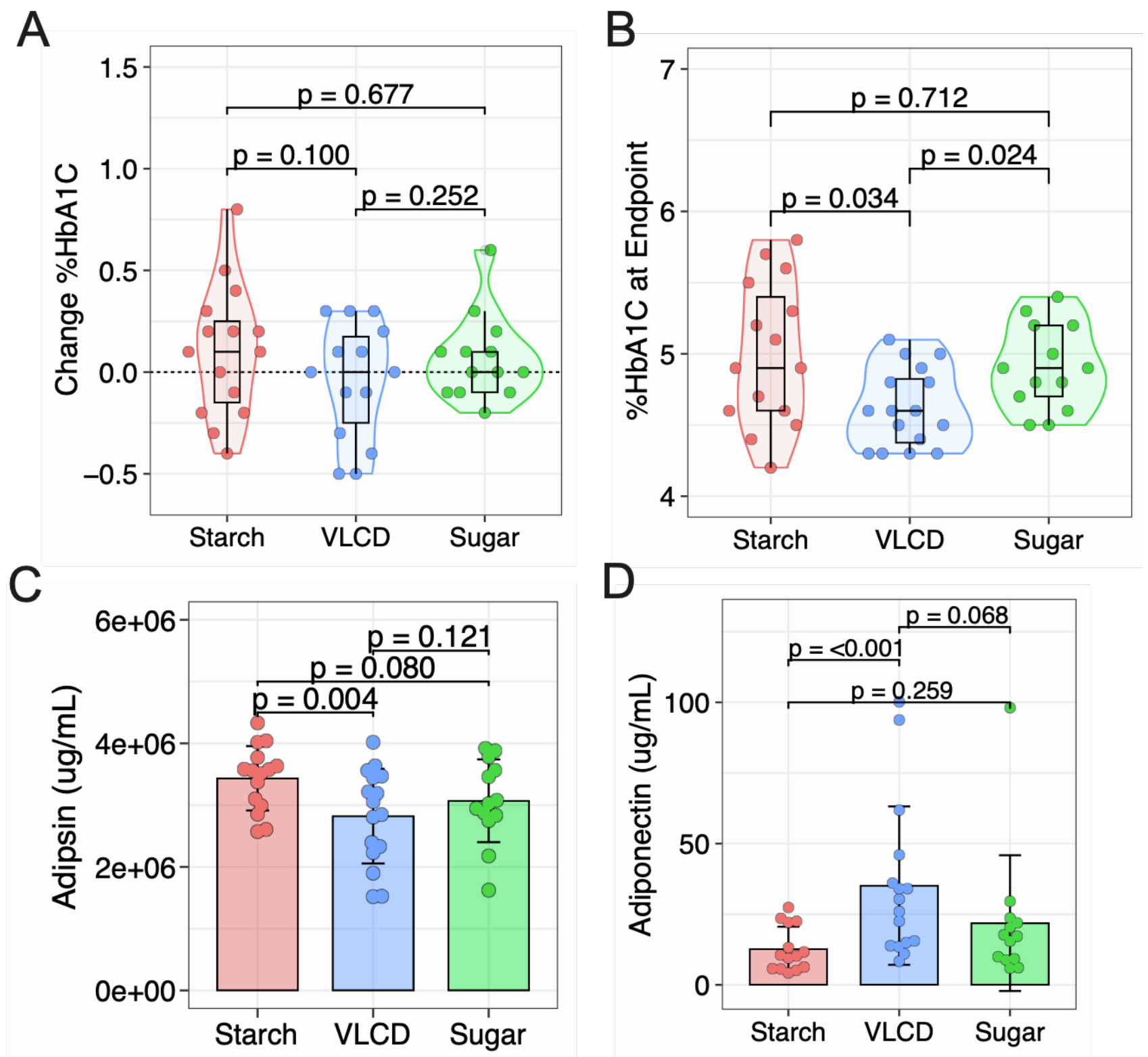

Supplementary Figure 2. Changes in HbA1c and absolute levels of HbA1c, adipsin and adiponectin in plasma at endpoint. Change (A) and absolute levels of HbA1c (B), adipsin (C) and adiponectin (D) at endpoint. Comparisons in A-D were done with Wilcoxon's test. For boxplots in A and B, horizontal bars within boxes represent medians. Tops and bottoms of boxes represent 25th and 75th percentiles, and vertical lines extend to the 1.5x interquartile range. Error bars represent standard deviation (SD). Statistical significance was defined as $p<0.05$.

Figure S3

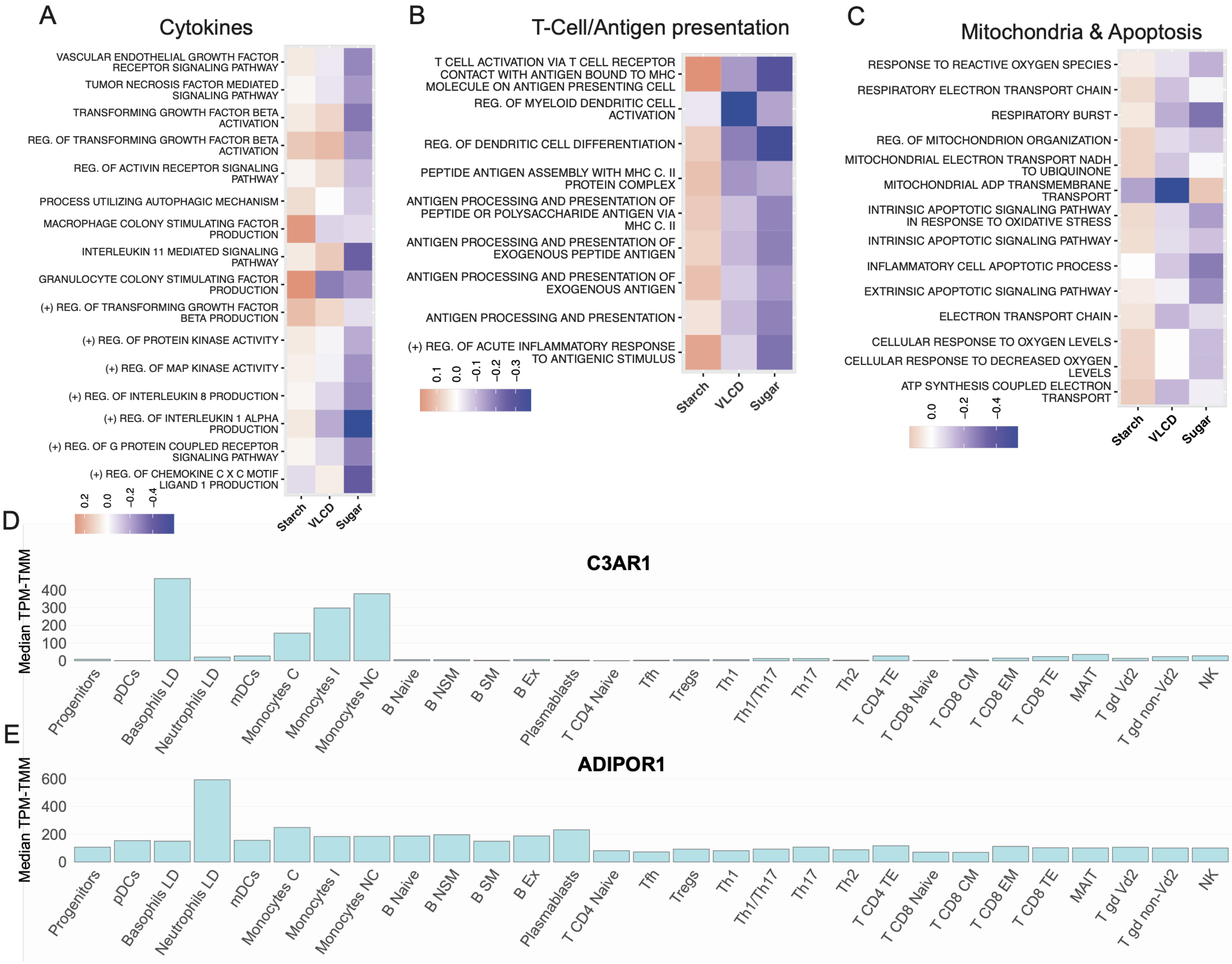
A
Cytokines
VASCULAR ENDOTHELIAL GROWTH FACTOR RECEPTOR SIGNALING PATHWAY
TUMOR NECROSIS FACTOR MEDIATED SIGNALING PATHWAY
TRANSFORMING GROWTH FACTOR BETA ACTIVATION
REG. OF TRANSFORMING GROWTH FACTOR BETA ACTIVATION
REG. OF ACTIVIN RECEPTOR SIGNALING PATHWAY
PROCESS UTILIZING AUTOPHAGIC MECHANISM
MACROPHAGE COLONY STIMULATING FACTOR PRODUCTION
INTERLEUKIN 11 MEDIATED SIGNALING PATHWAY
GRANULOCYTE COLONY STIMULATING FACTOR PRODUCTION
(+) REG. OF TRANSFORMING GROWTH FACTOR BETA PRODUCTION
(+) REG. OF PROTEIN KINASE ACTIVITY
(+) REG. OF MAP KINASE ACTIVITY
(+) REG. OF INTERLEUKIN 8 PRODUCTION
(+) REG. OF INTERLEUKIN 1 ALPHA PRODUCTION
(+) REG. OF G PROTEIN COUPLED RECEPTOR SIGNALING PATHWAY
(+) REG. OF CHEMOKINE C X C MOTIF LIGAND 1 PRODUCTION
0.2 0.0 −0.2 −0.4
Starch VLCD Sugar
B
T-Cell/Antigen presentation
T CELL ACTIVATION VIA T CELL RECEPTOR CONTACT WITH ANTIGEN BOUND TO MHC MOLECULE ON ANTIGEN PRESENTING CELL
REG. OF MYELOID DENDRITIC CELL ACTIVATION
REG. OF DENDRITIC CELL DIFFERENTIATION
PEPTIDE ANTIGEN ASSEMBLY WITH MHC C. II PROTEIN COMPLEX
ANTIGEN PROCESSING AND PRESENTATION OF PEPTIDE OR POLYSACCHARIDE ANTIGEN VIA MHC C. II
ANTIGEN PROCESSING AND PRESENTATION OF EXOGENOUS PEPTIDE ANTIGEN
ANTIGEN PROCESSING AND PRESENTATION OF EXOGENOUS ANTIGEN
ANTIGEN PROCESSING AND PRESENTATION
(+) REG. OF ACUTE INFLAMMATORY RESPONSE TO ANTIGENIC STIMULUS
0.1 0.0 −0.1 −0.2 −0.3
Starch VLCD Sugar
C
Mitochondria & Apoptosis
RESPONSE TO REACTIVE OXYGEN SPECIES
RESPIRATORY ELECTRON TRANSPORT CHAIN
RESPIRATORY BURST
REG. OF MITOCHONDRION ORGANIZATION
MITOCHONDRIAL ELECTRON TRANSPORT NADH TO UBIQUINONE
MITOCHONDRIAL ADP TRANSMEMBRANE TRANSPORT
INTRINSIC APOPTOTIC SIGNALING PATHWAY IN RESPONSE TO OXIDATIVE STRESS
INTRINSIC APOPTOTIC SIGNALING PATHWAY
INFLAMMATORY CELL APOPTOTIC PROCESS
EXTRINSIC APOPTOTIC SIGNALING PATHWAY
ELECTRON TRANSPORT CHAIN
CELLULAR RESPONSE TO OXYGEN LEVELS
CELLULAR RESPONSE TO DECREASED OXYGEN LEVELS
ATP SYNTHESIS COUPLED ELECTRON TRANSPORT
0.0 −0.2 −0.4
Starch VLCD Sugar
D
C3AR1
Median TPM-TMM
0 100 200 300 400
Progenitors pDCs Basophils LD Neutrophils LD mDCs Monocytes C Monocytes I Monocytes NC B Naive B NSM B SM B Ex Plasmablasts T CD4 Naive Tfh Tregs Th1 Th1/Th17 Th17 Th2 T CD4 TE T CD8 Naive T CD8 CM T CD8 EM T CD8 TE MAIT T gd Vd2 T gd non-Vd2 NK
E
ADIPOR1
Median TPM-TMM
0 200 400 600
Progenitors pDCs Basophils LD Neutrophils LD mDCs Monocytes C Monocytes I Monocytes NC B Naive B NSM B SM B Ex Plasmablasts T CD4 Naive Tfh Tregs Th1 Th1/Th17 Th17 Th2 T CD4 TE T CD8 Naive T CD8 CM T CD8 EM T CD8 TE MAIT T gd Vd2 T gd non-Vd2 NK

Supplementary Figure 3. Whole-blood transcriptomic changes in cytokines, antigen presentation and mitochondria related pathways after randomization to HC-Starch, HC-Sugar or second VLCD. A. Heatmap showing changes between END and START in the GSVA scoring of pathways related to cytokine signaling and secretion (A). Heatmap showing changes between END and START in the GSVA scoring of pathways related to T cell activity and antigen presentation (B). Heatmap showing changes between END and START in the GSVA scoring of pathways related to mitochondria and regulation of apoptosis (C). Expression of C3AR1 (D) and ADIPOR1 (E) from the public database ABIS. Heatmaps in A, B and C, were made using the mean value for the change (END-START) of the GSVA pathways scores. Progenitors (CD34+ hematopoietic progenitor cells); pDCs (plasmacytoid dendritic cells); Basophils LD (low-density basophils); Neutrophils LD (low-density neutrophils); mDCs (myeloid dendritic cells); Monocytes C (classical monocytes); Monocytes I (intermediate monocytes); Monocytes NC (non-classical monocytes); B Naive (naive B cells); B NSM (non-switched memory B cells); B SM (switched memory B cells); B Ex (exhausted B cells); Plasmablasts; T CD4 Naive (naive CD4 T cells); Tfh (T follicular helper cells); Tregs (T regulatory cells); Th1 (T helper 1 cells); Th1/Th17 (T helper 1/17 transitional cells); Th17 (T helper 17 cells); Th2 (T helper 2 cells); T CD4 TE (terminal effector CD4 T cells); T CD8 Naive (naive CD8 T cells); T CD8 CM (central memory CD8 T cells); T CD8 EM (effector memory CD8 T cells); T CD8 TE (terminal effector CD8 T cells); MAIT (mucosal-associated invariant T cells); T gd Vd2 (Vδ2 γδ T cells); T gd non-Vd2 (non-Vδ2 γδ T cells); NK (natural killer cells).

Figure S4

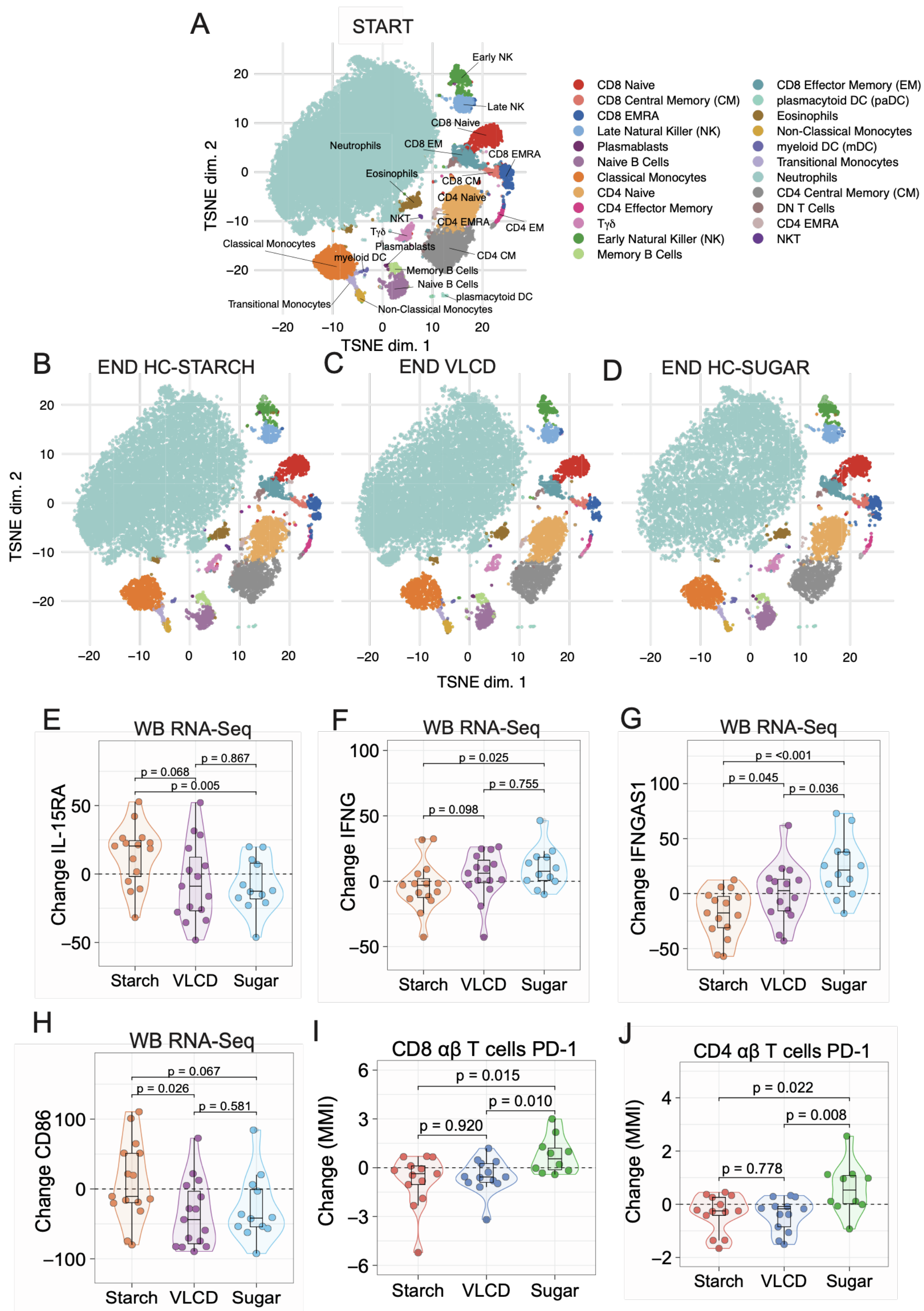

Supplementary Figure 4. Whole blood RNA-Seq and CyTOF analysis show distinct changes in leukocytes following randomization to diets with different carbohydrate composition after VLCD. A-D. A. T-SNE analysis of CyTOF whole-blood populations after VLCD during residential phase (A), and at the end of randomization to HC-Starch (B), VLCD (C), and HC-Sugar (D). **E-G.** Comparison of changes in IL-15(E) and IFNG (F), IFNGAS1(G) CD86 (H) expression in WB-RNAseq. I-J. Comparison of changes in PD1 expression in CD8αβ T cells (I) and CD4αβ T cells (J). Comparisons in E-J were made by subtracting the values from the START to the END (Change), followed by a Kruskal-Wallis' and Wilcoxon's test of the difference. For boxplots in E-J, horizontal bars within boxes represent medians. Tops and bottoms of boxes represent 25th and 75th percentiles, and vertical lines extend to the 1.5x interquartile range. Error bars represent standard deviation (SD). Statistical significance was defined as $p<0.05$. MMI is median metal intensity.

Figure S5

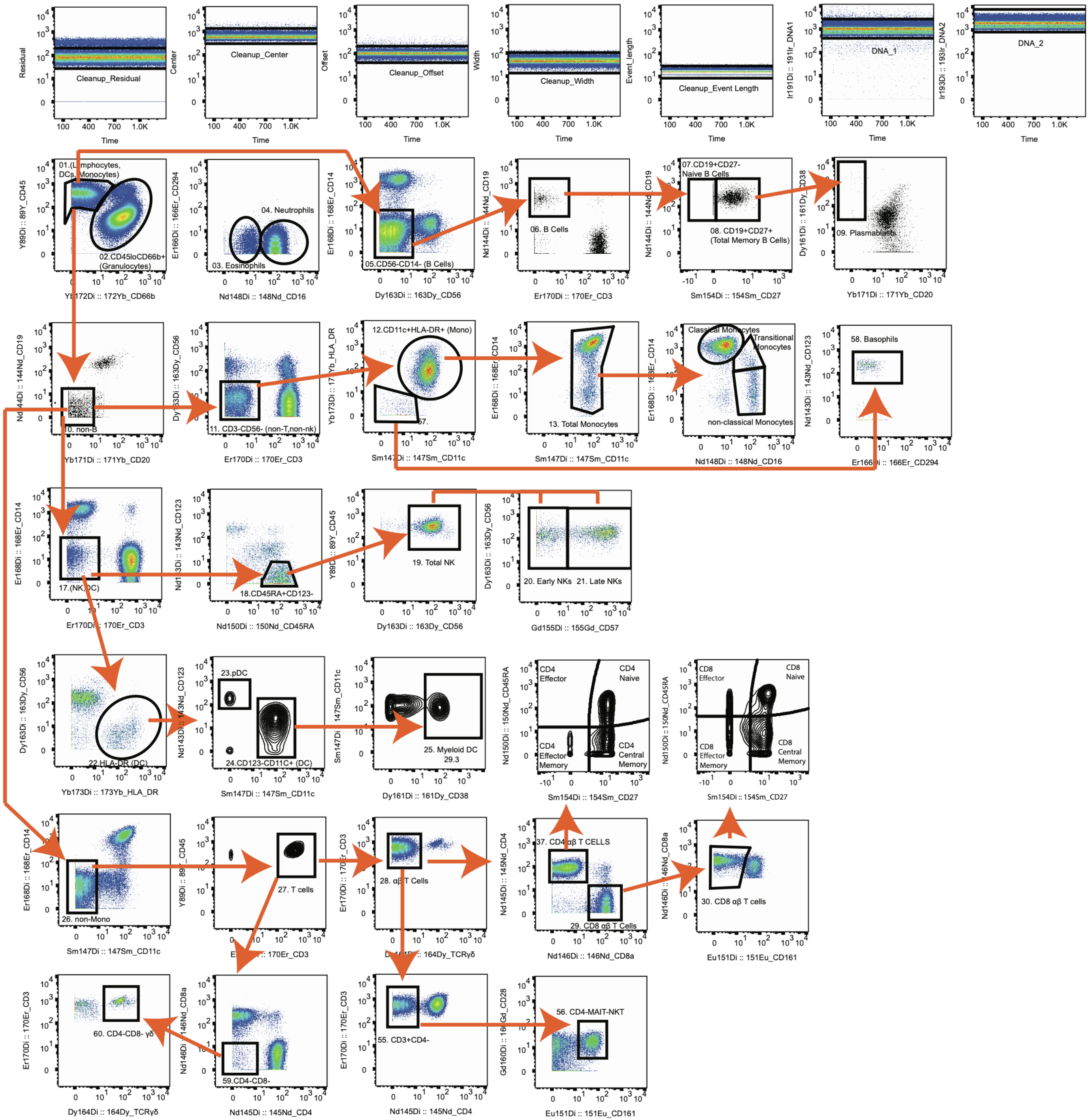

Supplementary Figure 5. Gating strategy for the whole blood CyTOF. MMI is median metal intensity

Figure S6

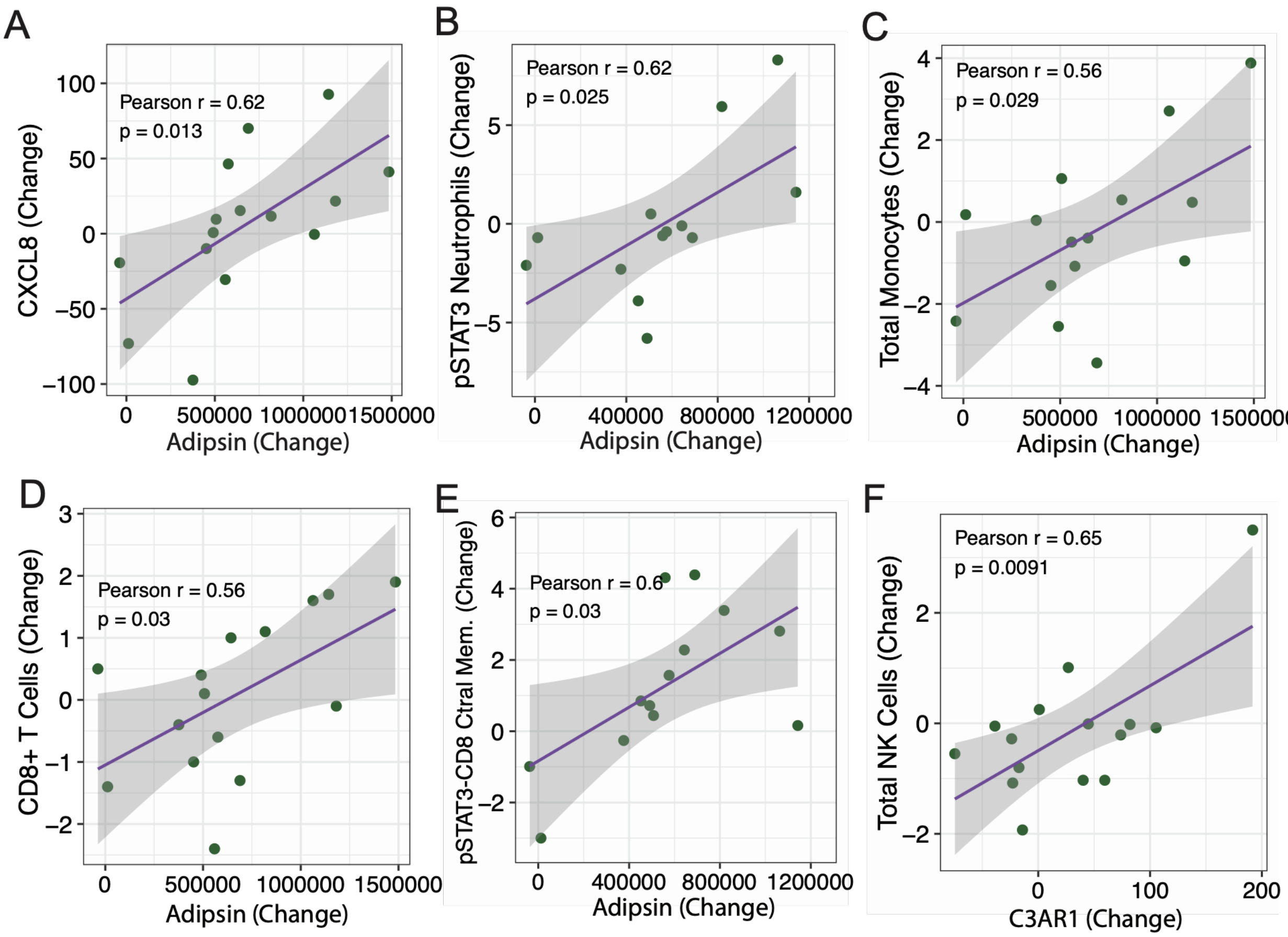

Supplementary Figure 6. Multimodal correlation analysis in HC-Starch group. (A) Correlation between change in CXCL8 transcript expression and change in plasma adipsin concentration in the HC-Starch group. (B–E) Correlations between change in plasma adipsin and change in p-STAT3 in neutrophils (B), total monocytes (%) (C), CD8+ T cells (%) (D), and p-STAT3 in central memory CD8+ T cells by CyTOF (E). (F) Correlation between change in total NK cell number and change in C3AR1 transcript expression. Changes are defined as END - START. Pearson's correlation coefficient was used.

Figure S7

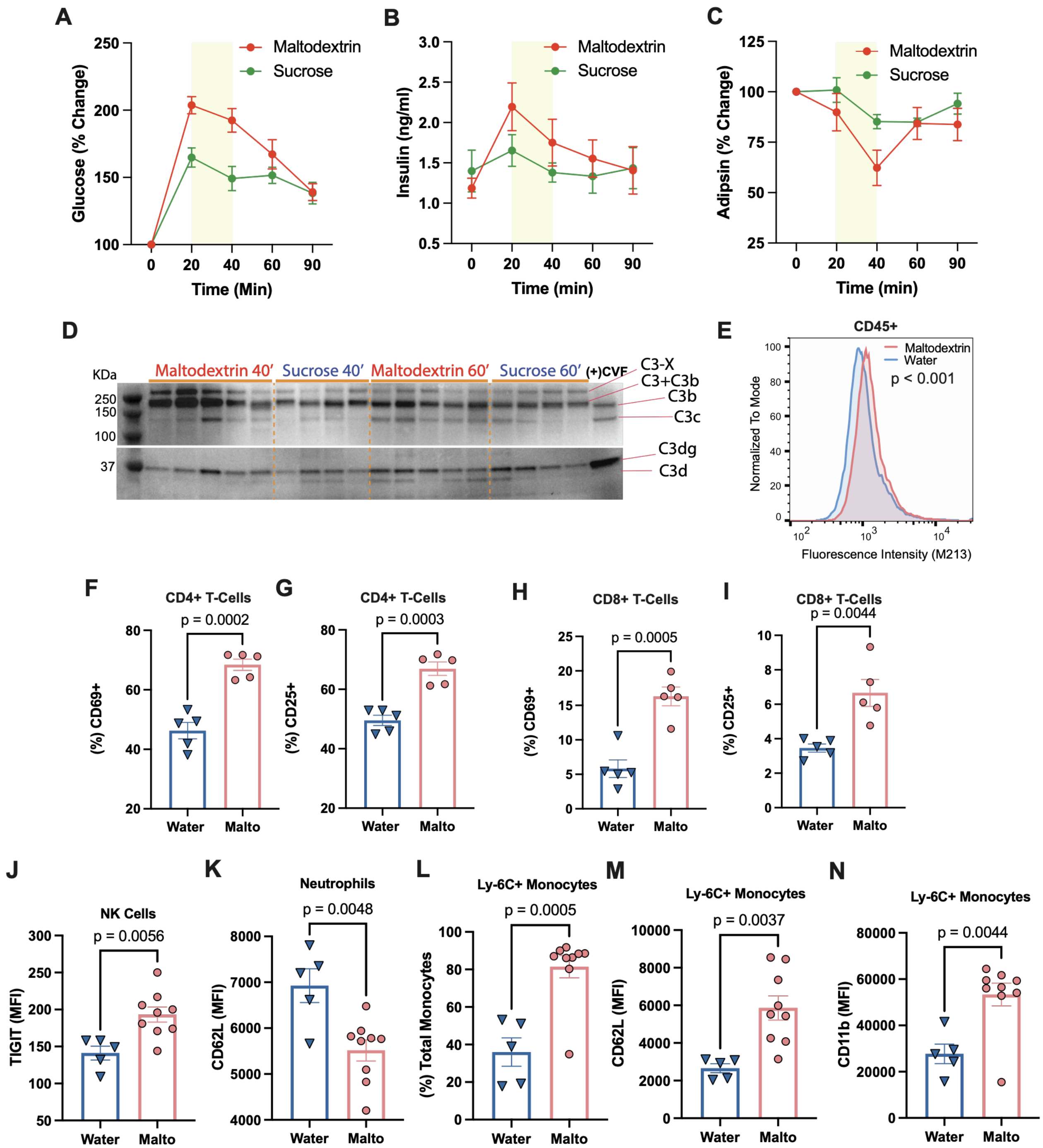

Supplementary Figure 7. Maltodextrin leads to complement and immune activation in mice. A-C. Serum levels of glucose (A), insulin (B), and adipsin (C) following an oral challenge with a 200 mg/ml solution of maltodextrin or sucrose. **D.** Western blot analysis showing C3 and its degradation fragments at 40 and 60 minutes after oral gavage with maltodextrin or sucrose. **E.** Histogram depicting the fluorescence intensity of C3 and its degradation fragments on the surface of CD45+ cells, assessed using the M213 antibody in mice challenged with maltodextrin or water. **F-G** Percentage of CD69+ (F) and CD25+ (G) CD4+ T cells one hour after oral gavage with maltodextrin or water. **H-I.** Percentage of CD69+ (H) and CD25+ (I) CD8+ T cells one hour after oral gavage with maltodextrin or water. **J-K.** Levels of TIGIT in natural killer cells (J) and CD62L in neutrophils (K) two hours after oral gavage with maltodextrin or water. **L.** Proportion of Ly-6C+ monocytes two hours after oral gavage with maltodextrin or water. **M-N.** Median expression levels of CD62L (M) and CD11b (N) in Ly-6C+ monocytes two hours after oral gavage with maltodextrin or water. Statistical comparisons in panels F-N were performed using two-tailed unpaired t-tests. MFI is Median Fluorescence Intensity. Comparison (E) was done using the Mann-Whitney U test. Comparisons in F-N were done using two-tailed Student's T-Test. Error bars represent standard error of the mean (SEM). Statistical significance was defined as $p<0.05$

Figure S8

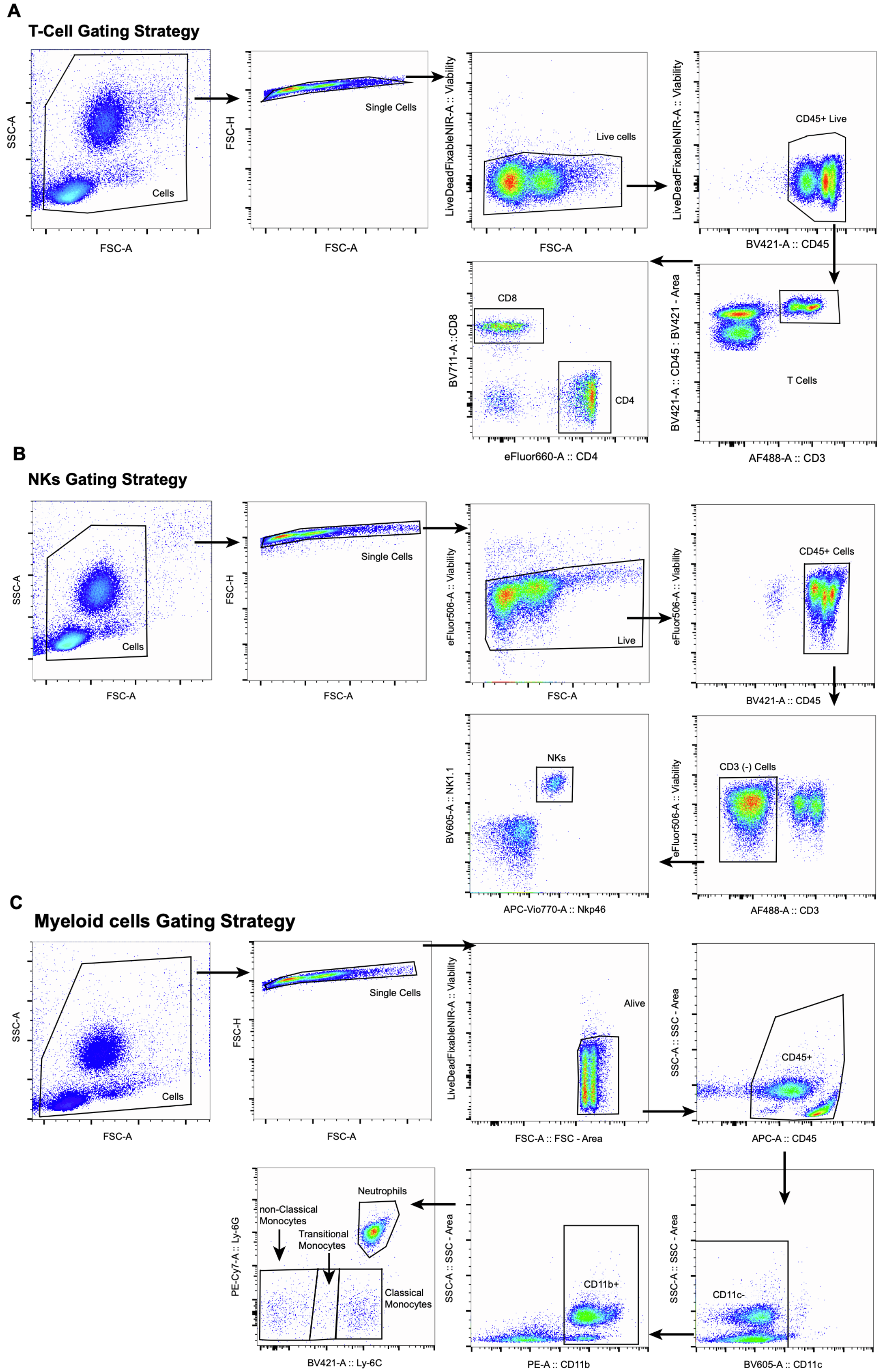

Supplementary Figure 8. Gating strategy for murine experiments. (A) Gating strategy for T-cell subsets, (B) natural killer cells (NKs), and (C) myeloid cells.

Supplementary Table 1

Supp.Table.1	**Cytokines**	**CyTOF (MDIPA)**	**CyTOF(Other)***	**RNA-SEQ**
Participants	44	40	36	42
Diet				
HC-Starch	15 (34%)	15 (38%)	13 (36%)	15 (36%)
HC-Sugar	13 (30%)	11 (28%)	10 (28%)	12 (29%)
VLCD	16 (36%)	14 (35%)	13 (36%)	15 (36%)
Sex				
Female	12 (27%)	10 (25%)	9 (25%)	11 (26%)
Male	32 (73%)	30 (75%)	27 (75%)	31 (74%)
BMI (Kg/m²)	26.8 (25.3, 31.0)	27.3 (25.3, 31.0)	27.7 (25.3, 31.0)	26.8 (25.2, 31.0)
Weight (Kg)	83 (77, 92)	84 (77, 96)	84 (77, 96)	83 (77, 94)
Age (Years)	35 (30, 42)	35 (28, 40)	35 (26, 40)	35 (29, 42)
Race				
Asian	1 (2.3%)	1 (2.5%)	1 (2.8%)	1 (2.4%)
Black	5 (11%)	5 (13%)	3 (8.3%)	5 (12%)
White	35 (80%)	31 (78%)	3 (8.3%)	33 (79%)
other	3 (6.8%)	3 (7.5%)	29 (81%)	3 (7.1%)

n (%); Median (IQR)

*Aditional antibodies (CD95, PD1, TIGIT, pSTAT3 (Tyr705), pNF-KB (p65, S529), p-S6(S235/S236)) were added to a subset of samples

Supplementary Table 1. Demographic characteristics of participants and analyses performed.

Supplementary Table 2

**Supp.Table 2**	HC-Starch			VLCD			HC-Sugar			Between Groups Comparisons (P-values)			
	Pre (Mean/SD)	Post (Mean/SD)	P-value	Pre (Mean/SD)	Post (Mean/SD)	P-value	Pre (Mean/SD)	Post (Mean/SD)	P-value	Overall	Starch vs Sugar	Starch vs VLCD	Sugar vs VLCD
Body mass index (kg/m2)	28.41 (4.07)	28.17 (3.86)	0.2860	28.66 (4.14)	27.84 (4.18)	0.0016	26.89 (3.03)	26.59 (3.01)	0.1680	0.0510	0.03	0.73	0.06
Glucose 2Hs (mg/dL)*	128.06 (18.40)	111.07 (17.24)	0.0050	80.86 (9.40)	80.21 (9.59)	1.0000	113.76 (12.20)	102 (13.74)	0.0820	0.0040	0.74	0.00	0.03
Fasting insulin (µIU/mL)	2.07 (1.28)	2.50 (1.34)	0.2100	2.62 (2.73)	3.66 (3.17)	0.2200	2.32 (1.48)	3.29 (1.32)	0.0470	0.9500	0.98	0.68	0.92
BHB (mM)	2.88 (1.22)	0.08 (0.03)	6.10E-05	2.70 (1.02)	1.09 (0.57)	0.0006	3.09 (1.92)	0.08 (0.02)	0.0002	0.0087	0.01	0.89	0.02
CRP (mg/L)	5.67 (10.35)	2.66 (3.47)	0.0098	5.73 (8.40)	3.28 (2.70)	0.0408	2.97 (4.70)	0.68 (0.42)	0.0033	0.3650	0.24	0.26	0.55
TG (mg/dL)	93.53 (30.75)	85.00 (47.27)	0.2440	84.81 (37.35)	54.19 (26.72)	0.0052	94.62 (46.90)	76.46 (30.34)	0.0843	0.2390	0.14	0.84	0.17

P-Value is from paired Wilcoxon Test. Overall is p-value from Kruskal Wallis Test, between group comparisons were done with Wilcoxon's Test

*CGM glucose 2 Hours After lunch, endpoint

Supplementary Table 2. Changes in metabolic parameters by diet group.

Supplementary Table 3

Supplementary Table 3 Cytokines	HC-Starch			VLCD (Mean)			HC-Sugar (Mean)			Between Groups Comparisons (P-values)			
	Start (Mean/SD)	End (Mean/SD)	P-value	Start (Mean/SD)	End (Mean/SD)	P-value	Start (Mean/SD)	End (Mean/SD)	P-value	Overall	Starch vs Sugar	Starch vs VLCD	Sugar vs VLCD
Adipsin (ug/mL)	2.77 (0.5)	3.43 (0.52)	1.83E-04	2.66 (0.69)	2.82 (0.77)	0.252	2.67 (0.53)	3.07 (0.67)	0.0017	0.0101	0.080	0.004	0.121
Adiponectin (ug/mL)	22.65 (21.72)	40.77 (109.04)	0.1070	28.08 (17.08)	35.12 (28.03)	0.231	29.86 (30.77)	21.85 (24.03)	0.0017	0.0142	0.274	0.049	0.006
IL-17A (pg/mL)	15.81 (20.31)	15.04 (20.25)	0.5760	8.84 (12.35)	9.31 (12.34)	0.149	47.07 (75.4)	39.18 (66.05)	0.0942	0.0551	0.182	0.101	0.041
IP-10 (pg/mL)	67.69 (32.29)	88.38 (34.64)	6.10E-05	65.6 (20.41)	75.86 (39.54)	0.597	272.62 (582.45)	124.73 (217.32)	0.2440	0.0643	0.130	0.021	0.589
IL-18 (pg/mL)	38.75 (45.27)	28.49 (29.98)	0.0215	18.55 (14.03)	17.47 (14.35)	0.222	179.17 (412.01)	223.91 (744.63)	0.2730	0.0686	0.650	0.021	0.144
GRO-α (pg/mL)	41.86 (29.78)	37.71 (27.89)	0.0181	29.74 (22.4)	29.58 (25.9)	0.632	38.2 (31.23)	29.51 (21.72)	0.0105	0.0751	0.413	0.247	0.017
IL-7 (pg/mL)	8.03 (9.23)	6.93 (6.85)	0.0479	8.93 (16.91)	9.08 (16.6)	0.980	28.43 (81.02)	14.91 (35.71)	0.1270	0.0838	1.000	0.030	0.121
Eotaxin (pg/mL)	38.8 (31.52)	35.01 (22.92)	0.2770	23.01 (11.92)	23.99 (12.22)	0.211	29.57 (8.81)	28.27 (9.88)	0.1910	0.0997	0.872	0.086	0.056
MIG-CXCL9 (ng/mL)	1.16 (0.62)	1.46 (1.03)	0.0946	1.21 (1.04)	1.01 (0.41)	0.348	2.1 (3.13)	1.33 (0.89)	0.3050	0.1040	0.065	0.078	0.846
IL-1α (pg/mL)	29.98 (43.16)	23.31 (27.27)	0.0637	18.64 (16.55)	18.9 (15.49)	0.551	186.4 (344.49)	157.64 (287.95)	0.2440	0.1320	0.821	0.040	0.188
IL-17E-IL-25 (ng/mL)	0.36 (0.39)	0.41 (0.56)	0.4560	0.32 (0.32)	0.3 (0.3)	0.572	1.28 (2.86)	1.16 (2.86)	0.0398	0.1430	0.107	0.953	0.076
IL-15 (pg/mL)	9.06 (5.18)	8.93 (4.62)	0.7980	13.06 (11.22)	13.45 (11.21)	0.443	50.83 (84.09)	40.49 (62.25)	0.0917	0.1480	0.117	0.984	0.072
TNF-β (ng/mL)	0.06 (0.1)	0.04 (0.06)	0.7540	0.02 (0.02)	0.02 (0.01)	0.478	1.17 (4.16)	1.01 (3.6)	0.0942	0.1550	0.496	0.236	0.062
IL-1RA (pg/mL)	13.49 (10.7)	7.7 (8.49)	0.0171	12.02 (15.14)	8.69 (13.35)	0.053	43.44 (64.28)	27.38 (46.81)	0.0171	0.1630	0.683	0.118	0.109
FLT-3L (pg/mL)	13.33 (5.35)	12.54 (4.38)	0.1510	11.91 (6.54)	11.84 (6.54)	1.000	22.95 (28.55)	19.74 (24.62)	0.0479	0.1700	0.467	0.213	0.083
IL-1β (pg/mL)	63.3 (98.06)	55.34 (80.71)	0.1620	24.92 (29.43)	25.18 (29.86)	0.887	129.04 (192.07)	111.43 (176.95)	0.0942	0.1730	0.712	0.118	0.119
TGF-α (pg/mL)	8.85 (7.65)	9.87 (10.66)	0.4780	6 (4.59)	6.9 (4.64)	0.222	30.66 (72.91)	48.86 (98.81)	0.2900	0.1980	0.140	0.118	0.861
MIP-1β (pg/mL)	49.98 (19.01)	50.76 (20.2)	0.5240	32.5 (10.68)	33.2 (10.91)	0.782	43.41 (19.05)	35.24 (16.48)	0.1100	0.2190	0.118	0.892	0.156
G-CSF (pg/mL)	28 (45.25)	26.83 (44.46)	0.8340	33.72 (34.73)	30.08 (31.46)	0.224	34.61 (74.57)	27.51 (67.23)	0.1960	0.2530	0.200	0.140	0.613
VEGF-A (pg/mL)	118.99 (92.42)	112.44 (97.99)	0.6390	184.88 (124.83)	190.52 (127.43)	0.980	258.04 (161.69)	228.23 (120.13)	0.0653	0.2670	0.201	0.800	0.144
IL-12p40 (pg/mL)	119.75 (171.66)	101.76 (103.17)	0.9340	97.04 (180.13)	65.37 (51.13)	0.551	323.59 (562.02)	168.25 (286.3)	0.1260	0.2970	0.222	0.984	0.148
FGF-2 (pg/mL)	183.07 (227.11)	164.43 (187.7)	0.1320	54.31 (29.3)	56.77 (25.8)	0.495	240.54 (306.56)	211.87 (258.47)	0.5880	0.3020	0.467	0.119	0.559
IL-8 (pg/mL)	9.6 (8.01)	8.12 (6)	0.1350	7.21 (3.51)	7.05 (2.99)	1.000	24.62 (35.53)	17.72 (21.15)	0.1270	0.3190	0.504	0.281	0.199
MIP-1α (pg/mL)	49.18 (13.96)	50.03 (21.58)	0.7200	50.69 (22.4)	50.25 (21.1)	0.900	97.05 (100.12)	87.47 (82.61)	0.1910	0.3250	0.274	0.599	0.184
RANTES (pg/mL)	2146.75 (472.15)	1953.05 (444.07)	0.3890	1866.44 (551.59)	1926.45 (483.58)	0.252	1623.05 (468.75)	1604.42 (470.64)	0.7870	0.3260	0.618	0.188	0.288
IL-17F (ng/mL)	0.01 (0.02)	0.02 (0.02)	0.8420	0.01 (0.01)	0.01 (0.01)	0.118	24.66 (88.85)	18.5 (66.55)	0.7840	0.4080	0.872	0.277	0.254
IL-10 (pg/mL)	2.92 (6.19)	3.27 (7.63)	0.4470	6.82 (9.41)	7.8 (11.66)	0.610	6.97 (15.21)	3.66 (10.99)	0.2950	0.4300	0.293	0.934	0.241
IL-27 (ng/mL)	1.84 (0.9)	1.77 (0.79)	0.2520	1.88 (0.92)	1.79 (0.74)	0.231	6.84 (19.1)	7.43 (22.17)	0.6850	0.5190	0.294	0.892	0.374
MCP-1 (pg/mL)	308.54 (89.96)	291.8 (95.85)	0.1070	291.04 (127.2)	278.19 (123.99)	0.298	323.09 (83.46)	288.32 (100.77)	0.0171	0.5540	0.717	0.545	0.288
TNF-α (ng/mL)	0.18 (0.27)	0.17 (0.2)	1.0000	0.08 (0.07)	0.08 (0.05)	1.000	1.09 (3.73)	1.03 (3.51)	0.2240	0.5650	0.413	1.000	0.329
IFN-α2 (pg/mL)	63.11 (71.9)	56.83 (53.57)	0.5290	43.12 (40.89)	37.82 (33.59)	0.142	189.62 (244.57)	149.75 (187.64)	0.2160	0.5910	0.407	0.890	0.368
PDGF-AA (pg/mL)	2656.04 (621.41)	2628.89 (555.96)	0.7620	2519.87 (595.14)	2521.11 (768.18)	0.433	2833.27 (732.95)	2891.32 (659.69)	0.5560	0.6290	0.786	0.495	0.398
sCD40L (ng/mL)	4.55 (1.94)	4.29 (2.06)	0.5610	3.48 (1.37)	3.83 (2.02)	0.495	4.58 (2.15)	4.59 (2.37)	1.0000	0.6400	0.717	0.358	0.650
IL-5 (pg/mL)	10.53 (23.64)	8.47 (15.08)	0.8040	4.05 (5.25)	4.4 (5.34)	0.528	6.78 (8.33)	5.11 (5.61)	0.1910	0.6480	0.496	0.861	0.398
IL-3 (pg/mL)	0.43 (1.14)	0.33 (0.87)	0.0935	0.16 (0.24)	0.16 (0.2)	0.850	0.54 (1.17)	0.43 (0.79)	0.8340	0.6530	0.337	0.585	0.860
Fractalkine (pg/mL)	276.64 (377.32)	263.28 (307.74)	0.7200	158.31 (130.69)	160.91 (117.08)	0.464	350.2 (726.91)	555.06 (1497.85)	0.5560	0.6630	0.496	0.423	0.914
PAI-total (ng/mL)	24.52 (5.51)	23.52 (6.33)	0.3590	26.6 (6.89)	26.01 (9.32)	0.597	26.62 (4.97)	26.57 (6.9)	0.7350	0.6650	0.316	0.654	0.914
IL-12p70 (pg/mL)	32.2 (85.28)	26.7 (56.98)	0.7240	4.67 (10.04)	4.27 (7.27)	1.000	37.85 (94.25)	44.42 (125.77)	0.3820	0.6870	0.519	1.000	0.415
M-CSF (pg/mL)	298.53 (854.64)	216.01 (536.99)	0.2090	88.77 (194.71)	78.04 (122.97)	0.979	104.71 (246.03)	96.88 (222.36)	0.1820	0.7150	0.945	0.502	0.497
Resistin (ng/mL)	36.24 (11.26)	37.93 (9.15)	0.3300	34.11 (8.76)	34.67 (10.8)	0.940	37.39 (13.93)	38 (12.7)	0.6350	0.7340	0.586	0.470	0.948
IFN-γ (pg/mL)	11.58 (23.63)	9.63 (15.92)	0.5240	2.17 (3.91)	2.04 (2.54)	0.887	18.13 (39.9)	23.88 (64.66)	0.9460	0.7410	0.717	0.545	0.559
IL-2 (pg/mL)	7.71 (16.65)	7.47 (12.5)	0.4100	3.2 (10.97)	1.16 (2.75)	0.625	33.51 (62.13)	16.28 (32.82)	0.5050	0.7640	0.872	0.451	0.661
IL-9 (pg/mL)	9.07 (7.38)	9.28 (6.45)	0.9000	8.75 (6.44)	9.63 (7.59)	0.108	14.9 (16.27)	14.55 (14.84)	0.9690	0.7780	0.908	0.464	0.676
Lipocalin-2 (ng/mL)	388.84 (219.12)	382.66 (164.05)	0.9340	313.41 (121.89)	345.61 (191.4)	0.821	383.3 (189.78)	408.17 (230.56)	0.4550	0.8180	0.525	0.770	0.812
MCP-3 (ng/mL)	0.04 (0.04)	0.04 (0.04)	0.6390	0.02 (0.01)	0.02 (0.01)	0.782	0.96 (3.39)	1.61 (5.74)	0.8930	0.8220	0.555	0.861	0.693
EGF (pg/mL)	75.6 (71.66)	78.76 (81.78)	0.6790	66.35 (35.51)	76.04 (37.55)	0.375	84.94 (47.15)	81.1 (41.32)	0.8390	0.8320	0.683	0.861	0.589
PDGF-AB-BB (ng/mL)	23.9 (3.78)	23.9 (4.45)	0.5240	23.53 (2.41)	23.97 (2.6)	0.074	25.13 (3.11)	25.66 (2.75)	0.4100	0.8540	0.751	0.572	1.000
IL-6 (pg/mL)	1.86 (2.43)	1.66 (2.3)	0.0125	2.16 (2.87)	1.18 (0.91)	0.044	2.04 (3.2)	3.48 (9.97)	0.1270	0.8690	0.927	0.664	0.682
MDC (pg/mL)	793.84 (295.03)	835.94 (298.89)	0.1210	916.04 (435.86)	890.93 (289.3)	1.000	810.83 (387.57)	834.74 (348.93)	0.5420	0.8900	0.650	0.830	0.619
IL-13 (ng/mL)	0.23 (0.33)	0.22 (0.26)	0.8020	0.12 (0.11)	0.12 (0.1)	0.842	3.36 (11.88)	2.22 (7.73)	0.9460	0.9220	0.964	0.752	0.746
IL-22 (pg/mL)	153.24 (205.59)	138.32 (153.99)	0.8340	79.75 (60.34)	79.81 (54.01)	0.932	39.61 (35.81)	45.41 (43.26)	0.7560	0.9310	0.714	0.859	0.889
IL-4 (pg/mL)	4.01 (8.68)	3.3 (5.6)	0.2630	3.01 (3.47)	2.89 (2.93)	0.980	3.17 (7.03)	2.89 (6.16)	0.4970	0.9520	0.872	0.937	0.559
GM-CSF (pg/mL)	190.94 (380.09)	164.12 (275.95)	0.4890	93.89 (193.89)	84.81 (159.18)	0.478	546.57 (1756.6)	559.16 (1820.98)	0.6250	0.9590	0.818	0.892	0.843
Leptin (pg/mL)	954.13 (741.19)	1313.66 (884.38)	0.0302	1232.69 (1087.31)	1729 (1380.42)	0.001	1162.08 (1207.03)	1825.07 (2019.32)	0.0640	0.9930	0.943	0.949	0.980

P-Value is from paired Wilcoxon Test. Overall is p-value from Kruskal Wallis Test, *Between Group Comparisons* were done with Wilcoxon's Test

Supplementary Table 3. Plasma cytokine concentrations and changes by diet group.

Supplementary Table 4

Supplemantary Table 4 GO:BP Pathways	HC-Starch Pre (Mean/SD)	HC-Starch Post (Mean/SD)	HC-Starch P-value	VLCD (Mean) Pre (Mean/SD)	VLCD (Mean) Post (Mean/SD)	VLCD (Mean) P-value	HC-Sugar (Mean) Pre (Mean/SD)	HC-Sugar (Mean) Post (Mean/SD)	HC-Sugar (Mean) P-value	Between Groups Comparisons: Starch vs Sugar	Starch vs VLCD	Sugar vs VLCD	Overall P-value	Overall P-adj.value
GOBP_PURINE_CONTAINING_COMPOUND_CATABOLIC_PROCESS	0.05 (0.13)	-0.01 (0.13)	0.048	-0.03 (0.16)	-0.01 (0.18)	0.804	-0.07 (0.09)	0.08 (0.11)	0.001	9.39E-05	0.148	0.028	0.000687	0.0401
GOBP_POSITIVE_REGULATION_OF_PROTEIN_SERINE_THREONINE_KINASE_ACTIVITY	-0.07 (0.14)	-0.01 (0.16)	0.303	0.01 (0.2)	-0.01 (0.19)	0.762	0.13 (0.14)	-0.07 (0.13)	0.001	0.00069	0.202	0.015	0.00135	0.0401
GOBP_POSITIVE_REGULATION_OF_MAP_KINASE_ACTIVITY	-0.07 (0.15)	-0.02 (0.17)	0.359	0.01 (0.22)	-0.02 (0.19)	0.762	0.17 (0.14)	-0.08 (0.14)	0.001	0.003	0.436	0.004	0.00189	0.0401
GOBP_POSITIVE_REGULATION_OF_CARBOHYDRATE_METABOLIC_PROCESS	-0.13 (0.16)	0 (0.19)	0.003	-0.01 (0.19)	0.03 (0.14)	0.33	0.1 (0.17)	0 (0.14)	0.016	0.000312	0.436	0.042	0.00248	0.0401
GOBP_RESPONSE_TO_GRAM_POSITIVE_BACTERIUM	-0.26 (0.38)	0.07 (0.48)	0.018	0.08 (0.56)	0.07 (0.53)	0.679	0.06 (0.4)	-0.35 (0.39)	0.007	0.000885	0.202	0.048	0.0027	0.0401
GOBP_GRANULOCYTE_COLONY_STIMULATING_FACTOR_PRODUCTION	-0.18 (0.46)	0.1 (0.38)	0.03	0.17 (0.37)	-0.13 (0.34)	0.022	0.18 (0.49)	-0.05 (0.58)	0.176	0.015	0.004	0.755	0.00345	0.0401
GOBP_TORC1_SIGNALING	-0.09 (0.16)	0.05 (0.18)	0.015	-0.03 (0.2)	-0.01 (0.18)	0.934	0.07 (0.2)	-0.03 (0.13)	0.012	0.001	0.146	0.167	0.00478	0.0401
GOBP_INTERLEUKIN_11_MEDIATED_SIGNALING_PATHWAY	-0.09 (0.42)	-0.03 (0.39)	0.679	-0.01 (0.32)	0.14 (0.39)	0.454	0.17 (0.36)	-0.21 (0.19)	0.001	0.008	0.806	0.008	0.00526	0.0401
GOBP_POSITIVE_REGULATION_OF_PROTEIN_KINASE_ACTIVITY	-0.06 (0.15)	0 (0.18)	0.421	0.02 (0.21)	0 (0.2)	0.934	0.09 (0.16)	-0.09 (0.14)	0.001	0.004	0.367	0.024	0.00526	0.0401
GOBP_POSITIVE_REGULATION_OF_GLYCOGEN_METABOLIC_PROCESS	-0.16 (0.25)	0 (0.31)	0.135	0.09 (0.18)	0.04 (0.23)	0.489	0.1 (0.26)	-0.09 (0.26)	0.002	0.003	0.111	0.152	0.00582	0.0401
GOBP_ELECTRON_TRANSPORT_CHAIN	-0.04 (0.12)	0.02 (0.14)	0.095	0.08 (0.2)	-0.06 (0.13)	0.007	0 (0.14)	-0.06 (0.11)	0.204	0.048	0.006	0.3	0.00599	0.0401
GOBP_MITOCHONDRIAL_ADP_TRANSMEMBRANE_TRANSPORT	0 (0.53)	-0.19 (0.55)	0.277	0.27 (0.49)	-0.26 (0.48)	0.002	0 (0.52)	0.13 (0.45)	0.11	0.137	0.137	0.003	0.00676	0.0401
GOBP_CELLULAR_RESPONSE_TO_OXYGEN_LEVELS	-0.08 (0.18)	0.02 (0.22)	0.107	0.01 (0.21)	0.01 (0.19)	0.978	0.08 (0.23)	-0.05 (0.18)	0.021	0.006	0.148	0.079	0.00751	0.0401
GOBP_T_HELPER_1_CELL_DIFFERENTIATION	-0.04 (0.19)	0.03 (0.2)	0.208	0.06 (0.2)	-0.03 (0.27)	0.252	0.06 (0.22)	-0.15 (0.21)	0.002	0.003	0.122	0.236	0.00767	0.0401
GOBP_CELLULAR_RESPONSE_TO_DECREASED_OXYGEN_LEVELS	-0.09 (0.18)	0.02 (0.21)	0.135	0.01 (0.2)	0.01 (0.19)	0.934	0.09 (0.22)	-0.05 (0.17)	0.021	0.006	0.217	0.07	0.00857	0.0401
GOBP_HIPPO_SIGNALING	-0.08 (0.21)	-0.02 (0.21)	0.561	-0.04 (0.24)	0.03 (0.26)	0.359	0.17 (0.23)	-0.06 (0.23)	0.009	0.011	1	0.011	0.00918	0.0401
GOBP_REGULATION_OF_ACUTE_INFLAMMATORY_RESPONSE_TO_ANTIGENIC_STIMULUS	-0.07 (0.26)	0.04 (0.26)	0.151	0.17 (0.26)	0.04 (0.22)	0.169	-0.01 (0.26)	-0.25 (0.23)	0.003	0.003	0.083	0.456	0.00926	0.0401
GOBP_REGULATION_OF_INOSITOL_PHOSPHATE_BIOSYNTHETIC_PROCESS	-0.16 (0.35)	-0.05 (0.27)	0.303	0.04 (0.44)	-0.07 (0.39)	0.72	0.29 (0.25)	-0.12 (0.32)	0.002	0.006	0.202	0.089	0.00932	0.0401
GOBP_RESPIRATORY_ELECTRON_TRANSPORT_CHAIN	-0.05 (0.13)	0.04 (0.16)	0.073	0.07 (0.22)	-0.05 (0.16)	0.035	-0.03 (0.15)	-0.05 (0.11)	0.733	0.112	0.007	0.277	0.00966	0.0401
GOBP_POSITIVE_REGULATION_OF_INTERLEUKIN_1_ALPHA_PRODUCTION	-0.12 (0.49)	-0.06 (0.35)	0.639	0.18 (0.45)	-0.01 (0.44)	0.252	0.23 (0.4)	-0.36 (0.41)	0.003	0.011	0.161	0.079	0.0105	0.0401
GOBP_REGULATION_OF_LIPID_TRANSPORT	-0.05 (0.14)	0.01 (0.12)	0.33	0.02 (0.17)	-0.01 (0.18)	0.804	0.08 (0.17)	-0.1 (0.13)	0.002	0.004	0.325	0.1	0.0105	0.0401
GOBP_LIPID_STORAGE	-0.05 (0.18)	-0.02 (0.2)	0.934	0.01 (0.22)	0.04 (0.18)	0.489	0.08 (0.19)	-0.12 (0.14)	0.009	0.013	0.838	0.013	0.0117	0.0401
GOBP_REGULATION_OF_MACROPHAGE_CHEMOTAXIS	-0.02 (0.28)	-0.04 (0.21)	1	0.02 (0.27)	0.03 (0.28)	0.72	0.14 (0.25)	-0.12 (0.23)	0.001	0.013	0.567	0.013	0.0117	0.0401
GOBP_REGULATION_OF_GRANULOCYTE_DIFFERENTIATION	-0.02 (0.28)	0.09 (0.27)	0.599	-0.07 (0.32)	0 (0.29)	0.489	0.14 (0.25)	-0.16 (0.21)	0.001	0.005	0.838	0.042	0.0118	0.0401
GOBP_EXTRINSIC_APOPTOTIC_SIGNALING_PATHWAY	-0.04 (0.19)	0 (0.18)	0.524	0.02 (0.24)	-0.01 (0.22)	0.89	0.11 (0.19)	-0.12 (0.17)	0.001	0.004	0.539	0.079	0.0119	0.0401
GOBP_POSITIVE_REGULATION_OF_CHEMOTAXIS	-0.06 (0.21)	-0.03 (0.2)	0.639	0.05 (0.26)	0.04 (0.26)	0.934	0.1 (0.18)	-0.14 (0.15)	0.003	0.011	0.806	0.028	0.0121	0.0401
GOBP_TRYPTOPHAN_TRANSPORT	-0.2 (0.29)	-0.02 (0.29)	0.055	0.09 (0.31)	-0.02 (0.28)	0.073	0.15 (0.3)	-0.02 (0.38)	0.301	0.037	0.015	0.548	0.0123	0.0401
GOBP_POSITIVE_REGULATION_OF_MONOCYTE_CHEMOTAXIS	-0.03 (0.27)	0.05 (0.25)	0.055	-0.01 (0.39)	0.01 (0.35)	0.978	0.11 (0.27)	-0.06 (0.31)	0.027	0.032	0.187	0.032	0.0143	0.0437
GOBP_REGULATION_OF_TRANSFORMING_GROWTH_FACTOR_BETA_ACTIVATION	-0.11 (0.34)	0.04 (0.31)	0.208	-0.12 (0.37)	0.07 (0.38)	0.135	0.18 (0.21)	-0.05 (0.19)	0.007	0.024	0.775	0.019	0.0144	0.0437
GOBP_REGULATION_OF_GLUCOSE_METABOLIC_PROCESS	-0.08 (0.16)	0.01 (0.16)	0.188	0.02 (0.16)	0 (0.13)	1	0.07 (0.16)	-0.03 (0.12)	0.021	0.013	0.187	0.125	0.0154	0.0437
GOBP_ATP_SYNTHESIS_COUPLED_ELECTRON_TRANSPORT	-0.07 (0.16)	0.05 (0.17)	0.064	0.08 (0.26)	-0.06 (0.18)	0.048	-0.04 (0.2)	-0.08 (0.12)	0.569	0.079	0.023	0.256	0.0159	0.0437
GOBP_POSITIVE_REGULATION_OF_TOLL_LIKE_RECEPTOR_4_SIGNALING_PATHWAY	-0.02 (0.35)	0.04 (0.36)	0.421	0.03 (0.39)	0.03 (0.33)	0.978	0.07 (0.23)	-0.19 (0.33)	0.007	0.011	0.653	0.055	0.0163	0.0437
GOBP_REGULATION_OF_ACTIVIN_RECEPTOR_SIGNALING_PATHWAY	-0.01 (0.24)	0.02 (0.19)	0.524	-0.07 (0.19)	0.01 (0.18)	0.135	0.09 (0.18)	-0.06 (0.22)	0.027	0.048	0.512	0.016	0.0166	0.0437
GOBP_REGULATION_OF_ADAPTIVE_IMMUNE_RESPONSE	-0.03 (0.15)	0 (0.14)	0.561	0.08 (0.2)	0 (0.18)	0.107	0.06 (0.17)	-0.09 (0.14)	0.003	0.013	0.183	0.183	0.0183	0.0437
GOBP_REGULATION_OF_MYELOID_DENDRITIC_CELL_ACTIVATION	0.11 (0.37)	0.08 (0.37)	0.89	0.18 (0.27)	-0.2 (0.27)	0	-0.02 (0.29)	-0.15 (0.35)	0.129	0.399	0.03	0.062	0.0196	0.0437
GOBP_RESPONSE_TO_DSRNA	-0.06 (0.2)	0.01 (0.2)	0.277	0.05 (0.2)	-0.02 (0.17)	0.188	0.12 (0.25)	-0.08 (0.23)	0.009	0.042	0.083	0.139	0.0196	0.0437
GOBP_T_CELL_ACTIVATION_VIA_T_CELL_RECEPTOR_CONTACT_WITH_ANTIGEN_BOUND_TO_	-0.06 (0.35)	0.13 (0.29)	0.151	0.08 (0.42)	-0.07 (0.45)	0.454	0.1 (0.43)	-0.2 (0.37)	0.016	0.016	0.122	0.323	0.0204	0.0437
GOBP_PROLINE_TRANSPORT	-0.2 (0.25)	-0.03 (0.3)	0.055	0.08 (0.22)	0.06 (0.34)	0.847	0.13 (0.25)	-0.01 (0.33)	0.077	0.011	0.222	0.236	0.0209	0.0437
GOBP_INFLAMMATORY_CELL_APOPTOTIC_PROCESS	-0.06 (0.18)	-0.05 (0.16)	1	0.09 (0.23)	-0.03 (0.23)	0.041	0.12 (0.24)	-0.15 (0.23)	0.005	0.026	0.217	0.1	0.0213	0.0437
GOBP_POSITIVE_REGULATION_OF_TRIGLYCERIDE_CATABOLIC_PROCESS	-0.12 (0.47)	0 (0.51)	0.421	0.12 (0.52)	0.03 (0.51)	0.679	0.15 (0.55)	-0.27 (0.44)	0.016	0.031	0.305	0.062	0.0216	0.0437
GOBP_TUMOR_NECROSIS_FACTOR_MEDIATED_SIGNALING_PATHWAY	-0.04 (0.16)	-0.01 (0.18)	0.762	0.05 (0.24)	-0.01 (0.24)	0.277	0.07 (0.21)	-0.16 (0.19)	0.005	0.022	0.345	0.079	0.0216	0.0437
GOBP_POSITIVE_REGULATION_OF_LEUKOCYTE_CHEMOTAXIS	-0.05 (0.21)	-0.01 (0.2)	0.561	0.04 (0.27)	0.03 (0.27)	0.847	0.09 (0.21)	-0.12 (0.22)	0.007	0.022	0.683	0.048	0.0217	0.0437
GOBP_L_ALPHA_AMINO_ACID_TRANSMEMBRANE_TRANSPORT	-0.09 (0.11)	0.02 (0.15)	0.055	0 (0.14)	-0.02 (0.13)	0.978	0.08 (0.09)	0.04 (0.12)	0.424	0.036	0.061	0.347	0.0218	0.0437
GOBP_REGULATION_OF_MITOCHONDRION_ORGANIZATION	-0.09 (0.12)	0.01 (0.13)	0.064	0.03 (0.16)	-0.04 (0.12)	0.169	0.07 (0.21)	-0.04 (0.13)	0.016	0.022	0.092	0.486	0.0243	0.0437
GOBP_PEPTIDE_ANTIGEN_ASSEMBLY_WITH_MHC_CLASS_II_PROTEIN_COMPLEX	-0.03 (0.36)	0.07 (0.37)	0.083	0.19 (0.4)	0.03 (0.34)	0.073	-0.04 (0.49)	-0.16 (0.41)	0.151	0.031	0.031	0.683	0.0244	0.0437
GOBP_RESPONSE_TO_REACTIVE_OXYGEN_SPECIES	-0.05 (0.16)	0 (0.15)	0.359	0.03 (0.17)	-0.03 (0.16)	0.421	0.09 (0.23)	-0.06 (0.18)	0	0.031	0.233	0.112	0.0253	0.0437
GOBP_ANTIGEN_PROCESSING_AND_PRESENTATION_OF_EXOGENOUS_ANTIGEN	-0.08 (0.25)	0.02 (0.24)	0.33	0.11 (0.28)	0.04 (0.25)	0.208	0.03 (0.33)	-0.12 (0.26)	0.002	0.026	0.146	0.236	0.0254	0.0437
GOBP_LEUKOCYTE_TETHERING_OR_ROLLING	-0.09 (0.22)	-0.01 (0.22)	0.252	0.02 (0.3)	0.04 (0.3)	0.89	0.11 (0.24)	-0.08 (0.23)	0.009	0.009	0.653	0.139	0.0255	0.0437
GOBP_INSULIN_LIKE_GROWTH_FACTOR_RECEPTOR_SIGNALING_PATHWAY	-0.06 (0.26)	0.03 (0.25)	0.639	0 (0.27)	-0.01 (0.31)	0.978	0.15 (0.22)	-0.09 (0.15)	0.007	0.016	0.512	0.112	0.0259	0.0437
GOBP_POSITIVE_REGULATION_OF_ACUTE_INFLAMMATORY_RESPONSE	-0.08 (0.27)	-0.01 (0.23)	0.303	0.11 (0.32)	0.11 (0.28)	1	0.01 (0.27)	-0.18 (0.26)	0.002	0.013	0.539	0.125	0.0259	0.0437
GOBP_MITOCHONDRIAL_ELECTRON_TRANSPORT_NADH_TO_UBIQUINONE	-0.05 (0.15)	0.05 (0.14)	0.064	0.06 (0.24)	-0.06 (0.17)	0.055	-0.03 (0.2)	-0.04 (0.1)	0.733	0.228	0.023	0.256	0.0262	0.0437
GOBP_REGULATION_OF_MACROPHAGE_ACTIVATION	-0.04 (0.21)	0 (0.2)	0.389	0.02 (0.27)	0.02 (0.22)	1	0.09 (0.16)	-0.09 (0.22)	0.016	0.022	0.653	0.07	0.0262	0.0437
GOBP_T_HELPER_1_TYPE_IMMUNE_RESPONSE	-0.02 (0.24)	0.03 (0.23)	0.303	0.08 (0.24)	-0.02 (0.3)	0.277	0.03 (0.25)	-0.15 (0.21)	0.009	0.019	0.146	0.373	0.0263	0.0437
GOBP_CELL_KILLING	-0.02 (0.17)	0.01 (0.19)	0.639	0.05 (0.26)	-0.03 (0.25)	0.169	0.09 (0.19)	-0.07 (0.22)	0.002	0.022	0.183	0.183	0.0272	0.0437
GOBP_LEUKOCYTE_MEDIATED_CYTOTOXICITY	-0.01 (0.18)	0.02 (0.21)	0.762	0.05 (0.26)	-0.05 (0.26)	0.083	0.11 (0.21)	-0.05 (0.24)	0.002	0.022	0.122	0.427	0.0273	0.0437
GOBP_PLASMA_CELL_DIFFERENTIATION	-0.12 (0.4)	-0.07 (0.34)	0.599	0.17 (0.36)	-0.02 (0.45)	0.018	0.13 (0.33)	-0.17 (0.31)	0.009	0.056	0.068	0.323	0.0288	0.0453
GOBP_CELLULAR_RESPONSE_TO_GLUCOSE_STARVATION	-0.1 (0.14)	0.01 (0.18)	0.041	-0.02 (0.22)	0.01 (0.15)	0.72	0.06 (0.2)	-0.03 (0.2)	0.077	0.022	0.233	0.233	0.0317	0.0481
GOBP_POSITIVE_REGULATION_OF_COMPLEMENT_ACTIVATION	-0.08 (0.47)	-0.02 (0.44)	0.679	0.18 (0.4)	-0.01 (0.44)	0.095	0.04 (0.39)	-0.29 (0.27)	0.009	0.056	0.092	0.277	0.0319	0.0481
GOBP_POSITIVE_REGULATION_OF_G_PROTEIN_COUPLED_RECEPTOR_SIGNALING_PATHWAY	-0.04 (0.27)	-0.01 (0.3)	0.72	0.09 (0.3)	0.01 (0.29)	0.303	0.07 (0.27)	-0.22 (0.21)	0.021	0.056	0.367	0.062	0.0325	0.0481
GOBP_GLUTAMINE_FAMILY_AMINO_ACID_CATABOLIC_PROCESS	-0.09 (0.2)	0 (0.19)	0.107	0.01 (0.16)	-0.08 (0.22)	0.277	0.16 (0.19)	0 (0.21)	0.034	0.042	0.068	0.792	0.0328	0.0481
GOBP_ANTIGEN_PROCESSING_AND_PRESENTATION_OF_PEPTIDE_OR_POLYSACCHARIDE_AN	-0.09 (0.32)	0 (0.32)	0.277	0.12 (0.27)	0.02 (0.25)	0.188	0.07 (0.34)	-0.11 (0.32)	0.012	0.056	0.122	0.217	0.0337	0.0481
GOBP_GLUTATHIONE_METABOLIC_PROCESS	-0.08 (0.17)	-0.06 (0.2)	0.679	0.09 (0.18)	-0.03 (0.2)	0.035	0.12 (0.14)	-0.02 (0.21)	0.007	0.048	0.061	0.867	0.0339	0.0481
GOBP_INTRINSIC_APOPTOTIC_SIGNALING_PATHWAY_IN_RESPONSE_TO_OXIDATIVE_STRESS	-0.09 (0.21)	0 (0.18)	0.188	0.07 (0.25)	-0.01 (0.23)	0.252	0.1 (0.28)	-0.1 (0.22)	0.043	0.048	0.159	0.217	0.0357	0.0493
GOBP_REGULATION_OF_DENDRITIC_CELL_DIFFERENTIATION	-0.06 (0.35)	0.03 (0.34)	0.524	0.14 (0.4)	-0.05 (0.37)	0.135	0.07 (0.39)	-0.29 (0.28)	0.009	0.036	0.111	0.548	0.0362	0.0493
GOBP_POSITIVE_REGULATION_OF_ACUTE_INFLAMMATORY_RESPONSE_TO_ANTIGENIC_STIMI	-0.09 (0.33)	0.06 (0.3)	0.083	0.19 (0.33)	0.12 (0.29)	0.33	-0.07 (0.36)	-0.28 (0.35)	0.043	0.031	0.111	0.683	0.0364	0.0493
GOBP_ANTIGEN_PROCESSING_AND_PRESENTATION_OF_EXOGENOUS_PEPTIDE_ANTIGEN	-0.05 (0.28)	0.03 (0.26)	0.454	0.14 (0.28)	0.04 (0.24)	0.107	0.04 (0.34)	-0.15 (0.26)	0.002	0.042	0.222	0.236	0.0407	0.0535
GOBP_POSITIVE_REGULATION_OF_CHEMOKINE_C_X_C_MOTIF_LIGAND_1_PRODUCTION	0.05 (0.58)	-0.03 (0.54)	0.679	0.01 (0.46)	0.06 (0.58)	0.489	0.15 (0.45)	-0.25 (0.43)	0.012	0.112	0.389	0.048	0.0407	0.0535
GOBP_REGULATION_OF_RAS_PROTEIN_SIGNAL_TRANSDUCTION	-0.04 (0.13)	-0.02 (0.17)	0.934	-0.02 (0.2)	0.01 (0.2)	0.679	0.1 (0.16)	-0.06 (0.12)	0.003	0.048	0.744	0.048	0.0422	0.0542
GOBP_T_CELL_PROLIFERATION	-0.05 (0.17)	0 (0.17)	0.561	0.06 (0.2)	-0.01 (0.18)	0.277	0.06 (0.18)	-0.1 (0.15)	0.003	0.026	0.25	0.25	0.0425	0.0542
GOBP_VASCULAR_ENDOTHELIAL_GROWTH_FACTOR_RECEPTOR_SIGNALING_PATHWAY	-0.13 (0.29)	-0.08 (0.26)	0.804	0.12 (0.29)	0.07 (0.26)	0.847	0.11 (0.23)	-0.16 (0.24)	0.007	0.048	1	0.07	0.0431	0.0542
GOBP_RESPONSE_TO_MOLECULE_OF_FUNGAL_ORIGIN	-0.17 (0.5)	-0.01 (0.46)	0.229	0.11 (0.51)	0.1 (0.49)	0.934	0.12 (0.48)	-0.13 (0.5)	0.012	0.026	0.412	0.228	0.044	0.0545
GOBP_REGULATION_OF_MYELOID_CELL_DIFFERENTIATION	-0.05 (0.21)	0 (0.19)	0.72	0.02 (0.2)	0 (0.19)	0.804	0.1 (0.22)	-0.1 (0.15)	0.009	0.056	0.683	0.079	0.0453	0.0551
GOBP_NADPH_OXIDATION	0.08 (0.35)	0.08 (0.37)	0.934	0.04 (0.49)	-0.22 (0.38)	0.121	-0.1 (0.41)	0.15 (0.45)	0.052	0.125	0.217	0.084	0.0457	0.0551
GOBP_INNATE_IMMUNE_RESPONSE	-0.05 (0.17)	-0.01 (0.16)	0.524	0.04 (0.23)	-0.01 (0.22)	0.33	0.08 (0.2)	-0.07 (0.2)	0.016	0.031	0.267	0.267	0.0477	0.0567
GOBP_REGULATION_OF_AUTOPHAGY	-0.07 (0.17)	0.02 (0.21)	0.33	-0.01 (0.23)	-0.02 (0.21)	0.934	0.07 (0.22)	-0.03 (0.18)	0.043	0.036	0.436	0.209	0.0502	0.0589
GOBP_MONOCYTE_ACTIVATION	-0.05 (0.36)	-0.03 (0.35)	0.762	0.01 (0.33)	0.02 (0.26)	0.89	0.14 (0.29)	-0.08 (0.27)	0.003	0.079	0.87	0.064	0.0517	0.0596
GOBP_POSITIVE_REGULATION_OF_TRANSFORMING_GROWTH_FACTOR_BETA_PRODUCTION	-0.12 (0.25)	0.05 (0.23)	0.03	-0.07 (0.27)	0.04 (0.24)	0.135	0.12 (0.16)	0.05 (0.24)	0.424	0.056	0.461	0.139	0.0525	0.0596
GOBP_POSITIVE_REGULATION_OF_INTERLEUKIN_8_PRODUCTION	-0.05 (0.26)	-0.02 (0.28)	0.72	0.07 (0.34)	-0.02 (0.33)	0.359	0.12 (0.33)	-0.15 (0.27)	0.005	0.064	0.345	0.154	0.0528	0.0596
GOBP_PROCESS_UTILIZING_AUTOPHAGIC_MECHANISM	-0.06 (0.17)	0.03 (0.21)	0.303	-0.01 (0.23)	-0.01 (0.21)	0.934	0.07 (0.2)	-0.05 (0.18)	0.007	0.048	0.412	0.228	0.0597	0.0665
GOBP_INTRINSIC_APOPTOTIC_SIGNALING_PATHWAY	-0.06 (0.16)	0.02 (0.13)	0.208	0.04 (0.17)	-0.03 (0.12)	0.208	0.07 (0.22)	-0.05 (0.14)	0.092	0.101	0.101	0.456	0.0673	0.0740
GOBP_T_CELL_MEDIATED_CYTOTOXICITY	-0.03 (0.18)	0.03 (0.19)	0.151	0.04 (0.27)	0 (0.23)	0.303	0.06 (0.22)	-0.04 (0.26)	0.009	0.056	0.206	0.683	0.0708	0.0769
GOBP_LEUCINE_METABOLIC_PROCESS	-0.01 (0.27)	0.07 (0.31)	0.599	0.09 (0.33)	-0.12 (0.2)	0.026	-0.08 (0.34)	-0.01 (0.37)	0.622	1	0.079	0.171	0.0748	0.0803
GOBP_MACROPHAGE_COLONY_STIMULATING_FACTOR_PRODUCTION	-0.18 (0.33)	0.09 (0.32)	0.083	0.05 (0.24)	-0.05 (0.26)	0.208	0.13 (0.39)	0.06 (0.47)	0.47	0.101	0.101	0.867	0.0786	0.0833
GOBP_ANTIGEN_PROCESSING_AND_PRESENTATION	-0.06 (0.22)	-0.01 (0.24)	0.639	0.09 (0.27)	0 (0.24)	0.135	0.08 (0.26)	-0.11 (0.24)	0.016	0.096	0.277	0.277	0.0804	0.0842
GOBP_RESPIRATORY_BURST	-0.11 (0.3)	-0.05 (0.29)	0.72	0.16 (0.38)	0 (0.34)	0.095	0.11 (0.34)	-0.18 (0.33)	0.001	0.096	0.25	0.25	0.0833	0.0862
GOBP_REGULATION_OF_LYMPHOCYTE_MEDIATED_IMMUNITY	-0.02 (0.12)	0.02 (0.12)	0.208	0.03 (0.17)	-0.01 (0.16)	0.454	0.05 (0.13)	-0.02 (0.14)	0.034	0.064	0.35	0.427	0.085	0.0870
GOBP_AMINO_ACID_TRANSMEMBRANE_TRANSPORT	-0.08 (0.09)	0.02 (0.15)	0.041	0.01 (0.13)	-0.02 (0.11)	0.804	0.07 (0.09)	0.02 (0.11)	0.301	0.074	0.35	0.456	0.0938	0.0949
GOBP_MONOSACCHARIDE_METABOLIC_PROCESS	-0.07 (0.18)	-0.04 (0.17)	0.679	0.07 (0.21)	-0.04 (0.15)	0.169	0.08 (0.17)	-0.09 (0.11)	0	0.124	0.326	0.373	0.108	0.1080

Supplementary Table 4. Changes in gene set variation analysis (GSVA) scores by diet group.

Supplementary Table 5

Supplementary Table 5<br>CyTOF	HC-Starch<br>Start<br>(Mean/SD)	End<br>(Mean/SD)	P-value	VLCD (Mean)<br>Start<br>(Mean/SD)	End<br>(Mean/SD)	P-value	HC-Sugar (Mean)<br>Start<br>(Mean/SD)	End<br>(Mean/SD)	P-value	Between Groups Comparisons (P-values)<br>Overall	Starch vs Sugar	Starch vs VLCD	Sugar vs VLCD
non-classical monocytes HLA-DR (Mean)	150.99 (56.52)	174.93 (61.59)	0.009	135.31 (62.22)	116.68 (46.74)	0.426	135.53 (60.98)	161.3 (53.71)	0.005	0.0084	0.917	0.006	0.009
CD4αβ PD1 (Mean)	3.99 (1.51)	3.65 (1.13)	0.273	4.34 (1.55)	3.94 (1.52)	0.059	4.36 (1.3)	4.96 (2.08)	0.049	0.0166	0.022	0.778	0.008
TCells PD1 (Mean)	4.04 (1.51)	3.6 (1.2)	0.146	4.44 (1.64)	4.07 (1.6)	0.094	4.38 (1.39)	5.01 (2.13)	0.105	0.0167	0.012	0.96	0.01
CD8αβ PD1 (Mean)	4.64 (2.55)	3.85 (1.79)	0.127	5.63 (3.45)	5.11 (3.05)	0.068	4.96 (2.15)	5.72 (2.67)	0.131	0.0183	0.015	0.92	0.01
CD4 central memory PD1 (Mean)	6.17 (1.82)	5.61 (1.46)	0.08	7.22 (1.82)	6.58 (1.9)	0.033	6.93 (1.84)	7.63 (2.8)	0.16	0.0202	0.012	0.88	0.015
Neutrophils pNF-kB (Mean)	66.8 (12.86)	74.17 (20.41)	0.127	80.37 (20.33)	69.37 (19.76)	0.013	76 (39.39)	74.86 (34.5)	0.846	0.022	0.137	0.006	0.264
Early NKs (% of Total NK)	42.57 (9.06)	47.22 (10.69)	0.004	54.34 (12.05)	55.48 (10.28)	0.272	50.25 (9.59)	52.01 (9.63)	0.005	0.0285	0.033	0.026	0.338
Late NKs (% of Total NK)	57.11 (8.98)	52.53 (10.63)	0.003	45.46 (12.09)	44.33 (10.3)	0.326	49.49 (9.74)	47.57 (10.09)	0.008	0.0317	0.048	0.026	0.262
CD4 Naive pNF-kB (Mean)	47.78 (15.51)	61.01 (23.45)	0.006	45.02 (15.72)	45.38 (14.99)	0.861	45.03 (24.51)	45.16 (23.9)	0.695	0.0319	0.025	0.026	0.879
Neutrophils pSTAT3 (Mean)	18.4 (4.84)	18.38 (4.95)	0.485	23.69 (4.36)	20.24 (4.21)	0.011	18.87 (4.18)	19.14 (4.36)	0.919	0.0336	0.71	0.043	0.02
Transitional monocytes CD38 (Mean)	24.13 (8.73)	30.75 (10.78)	0.005	23.12 (11.9)	22.74 (7.09)	0.583	23.12 (13.59)	26.94 (12.96)	0.054	0.0403	0.406	0.011	0.202
Myeloid DC pNF-kB (Mean)	65.6 (21.22)	83.43 (34.64)	0.048	74.38 (23.64)	67.69 (20.43)	0.216	64.42 (30.24)	65.18 (31.6)	1	0.0424	0.067	0.026	0.376
Classical monocytes pS6 (Mean)	3.01 (0.57)	3.25 (0.62)	0.34	2.84 (0.81)	2.35 (0.76)	0.04	3.03 (1.46)	2.9 (1.41)	0.322	0.0439	0.131	0.022	0.232
CD4αβ pNF-kB (Mean)	59.15 (17.71)	70.48 (22.72)	0.008	55.22 (18.42)	55.11 (17.15)	0.839	55.56 (29.7)	58.27 (30.32)	1	0.0481	0.044	0.034	0.733
CD4 Naive PD1 (Mean)	0.91 (0.82)	1.02 (0.84)	0.127	0.62 (0.21)	0.62 (0.24)	0.726	0.67 (0.3)	0.82 (0.36)	0.032	0.0653	0.687	0.101	0.025
CD8 Naive pNF-kB (Mean)	52.78 (18.33)	64.17 (24.21)	0.011	51.15 (17.84)	51.93 (16.82)	0.893	49.67 (25.82)	50.25 (26.77)	0.625	0.0654	0.025	0.081	0.976
non-classical monocytes CD38 (Mean)	10.95 (10.53)	15.75 (14.25)	0.007	11.08 (13.01)	8.59 (3.89)	1	11.41 (15.25)	14.46 (17.54)	0.032	0.074	0.474	0.029	0.166
Tcells pNF-kB (Mean)	59.78 (19.33)	70.78 (23.29)	0.014	55.6 (18.81)	55.75 (17.49)	0.839	55.52 (29.31)	58.36 (30.72)	1	0.0756	0.059	0.058	0.648
Late NKs pNF-kB (Mean)	48.61 (19.74)	60.92 (26.77)	0.006	47.82 (22.41)	48.55 (20.43)	1	43.78 (26.57)	44.51 (27.21)	0.695	0.083	0.036	0.091	1
Total monos pS6 (Mean)	3.6 (0.71)	3.85 (1.1)	0.944	3.42 (0.92)	2.83 (1.1)	0.048	3.49 (1.71)	3.22 (1.53)	0.16	0.0857	0.251	0.035	0.313
Classical monocytes pNF-kB (Mean)	65.79 (21.55)	83.4 (37.49)	0.033	72.44 (25.49)	69.8 (22.21)	0.78	67.78 (33.43)	69.36 (34.2)	0.77	0.0947	0.166	0.04	0.556
Total monos pS6 (Mean)	66.95 (21.56)	84.54 (37.9)	0.033	73.38 (26.22)	70.58 (22.69)	0.861	68.44 (34.05)	69.72 (34.63)	0.846	0.0999	0.131	0.05	0.577
non-classical monocytes pNF-kB (Mean)	72.81 (23.62)	91.25 (40.71)	0.027	75.95 (29.91)	74.55 (27.53)	1	73.12 (40.48)	71.94 (38.05)	0.922	0.104	0.067	0.081	1
CD8αβ pNF-kB (Mean)	56.89 (20.43)	67.42 (23.75)	0.011	54.18 (19.52)	55.23 (18.39)	0.889	52.16 (26.94)	54.25 (28.6)	1	0.107	0.058	0.096	0.975
Plasmablasts (% of Total Memory BCells)	1.4 (1.55)	2.92 (4.11)	0.073	1.83 (1.99)	1.37 (1.42)	0.391	2.78 (6.81)	1.26 (1.54)	0.898	0.11	0.113	0.063	0.647
Early NKs CD95 (Mean)	11.22 (3.12)	12.53 (3.68)	0.146	12.08 (3.92)	11.37 (3.43)	0.414	11.02 (4.61)	10.63 (4.6)	0.375	0.114	0.042	0.13	1
BCells CD95 (Mean)	5.97 (2.15)	8.37 (4.83)	0.08	6.52 (4.39)	5.71 (1.94)	0.839	5.48 (3.49)	4.81 (1.58)	0.557	0.118	0.067	0.101	0.927
Neutrophils pS6 (Mean)	6.18 (3.88)	6.32 (3.22)	0.946	5.16 (2.96)	3.6 (1.47)	0.057	5.61 (3.8)	5.87 (3.26)	0.432	0.119	0.976	0.101	0.067
T cells CD38 (Mean)	18.95 (5.87)	18.6 (5.03)	0.489	20.8 (5.23)	19.14 (4.77)	0.079	18.05 (4.91)	18.55 (5.04)	0.35	0.121	0.299	0.162	0.075
CD4 effector memory pS6 (Mean)	3.48 (0.92)	3.52 (0.79)	0.735	3.21 (0.97)	2.56 (0.74)	0.048	3.4 (1.63)	3.03 (1.43)	0.105	0.126	0.131	0.072	0.605
CD4 central memory pNF-kB (Mean)	66.08 (19.63)	76.46 (22.02)	0.008	62.04 (19.86)	61.98 (18.64)	0.946	61.52 (31.25)	65.72 (33.25)	0.695	0.13	0.115	0.081	0.648
αβT cells CD38 (Mean)	19.49 (6.14)	19.06 (5.12)	0.443	21.53 (5.56)	19.79 (5.05)	0.068	18.98 (5.2)	19.43 (5.08)	0.423	0.135	0.299	0.176	0.085
CD4 central memory pS6 (Mean)	3.43 (0.57)	3.49 (0.66)	0.893	3.08 (0.9)	2.51 (0.82)	0.039	3.34 (1.6)	3.05 (1.44)	0.105	0.136	0.166	0.081	0.41
CD4 effector PD1 (Mean)	4.33 (3.12)	2.75 (2.3)	0	3.11 (2.42)	2.57 (2.22)	0.497	6.96 (12.12)	8.15 (17.16)	0.625	0.136	0.03	0.264	0.738
CD8 central memory PD1 (Mean)	12.43 (6)	10.32 (3.99)	0.059	14.65 (4.12)	13.4 (3.67)	0.075	13.54 (5.47)	14.11 (5.74)	0.557	0.14	0.077	0.898	0.101
CD4 effector pS6 (Mean)	6.71 (3.23)	7.6 (3.5)	0.327	6.42 (2.84)	5.09 (2.34)	0.146	5.38 (4.55)	4.42 (2.48)	0.557	0.145	0.131	0.101	0.446
CD4 effector (% of Total CD4+ αβT Cells)	1.67 (2.98)	2.68 (1.94)	0.008	1.18 (2.24)	2.61 (3.96)	0.049	1.73 (2.27)	2.18 (1.78)	0.413	0.146	0.077	0.172	0.501
γδ-TCells CD38 (Mean)	7.03 (6.26)	8.2 (6.57)	0.03	5.14 (2.37)	4.93 (2.27)	0.715	4.65 (2.86)	5.07 (2.88)	0.126	0.152	0.134	0.093	0.647
Early NKs pSTAT3 (Mean)	3.38 (1.17)	4.26 (1.75)	0.068	3.66 (1.81)	3.48 (1.39)	0.735	3.55 (1.54)	2.86 (0.56)	0.375	0.161	0.072	0.243	0.446
CD4 effector memory PD1 (Mean)	6.17 (2.09)	5.65 (1.83)	0.273	7.23 (1.82)	6.49 (2.09)	0.048	7.09 (1.89)	8.14 (3.8)	0.432	0.162	0.208	0.448	0.077
Myeloid DC HLA.DR (Mean)	557.6 (215.83)	469.67 (147.39)	0.035	508.57 (160.46)	464.5 (154.54)	0.542	479 (88.13)	486 (108.7)	0.594	0.168	0.065	0.295	0.434
Myeloid DC TIGIT (Mean)	3.06 (2.23)	3.89 (2.48)	0.588	2.2 (1.23)	2.13 (1.39)	0.845	3.17 (1.44)	2.87 (2.35)	0.16	0.173	0.131	0.59	0.101
γδ-Tcells (% of T-Cells)	2.94 (2.15)	3.09 (2.14)	0.72	3.78 (2.25)	3.38 (1.91)	0.042	4.68 (4.9)	4.54 (5.18)	0.1	0.182	0.177	0.093	0.827
CD8 central memory pSTAT3 (Mean)	4.45 (1.28)	5.73 (2.37)	0.057	4.37 (2.18)	4.56 (2.38)	1	3.87 (1.31)	3.94 (1.08)	0.77	0.2	0.131	0.139	0.927
CD8 central memory pS6 (Mean)	3.46 (0.85)	3.5 (0.65)	0.944	3.15 (0.95)	2.54 (0.84)	0.022	3.17 (1.52)	2.92 (1.36)	0.16	0.202	0.277	0.118	0.343
Classical Monocytes TIGIT (Mean)	1.69 (1.6)	2.33 (1.55)	0.057	1.31 (0.87)	1.38 (1.11)	0.893	1.44 (1)	1.53 (0.89)	0.695	0.207	0.257	0.096	0.648
CD4 Naive CD95 (Mean)	7.24 (2.75)	9.85 (4.45)	0.048	6.12 (3.1)	6.36 (2.26)	0.455	6.75 (5.45)	6.09 (2.29)	0.922	0.209	0.148	0.169	0.522
MAIT/NKT (%of CD3+ CD4-)	15.86 (15.12)	16.17 (15.66)	0.572	15.55 (8.39)	14.98 (8.55)	0.326	17.59 (10.87)	18.93 (11.74)	0.265	0.215	0.406	0.172	0.163
CD8 Effector pNF-kB (Mean)	55.25 (21.82)	64.48 (23.76)	0.022	52.73 (22.48)	53.98 (21.94)	0.946	47.96 (25.5)	49.12 (26.72)	0.922	0.223	0.208	0.113	0.976
Late NK CD95 (Mean)	7.72 (2.16)	8.81 (3.37)	0.17	8.71 (2.85)	8.31 (2.39)	1	8.06 (3.21)	7.65 (2.7)	0.275	0.225	0.101	0.311	0.483
CD8 Naïve CD38 (Mean)	10.72 (3.44)	11.09 (3.32)	0.639	10.68 (3.21)	10.31 (3.48)	0.426	11.91 (4.4)	12.36 (4.67)	0.278	0.231	0.384	0.222	0.155
Early NK pNF-kB (Mean)	52.2 (22.26)	64.02 (28.34)	0.04	51.35 (23.34)	49.18 (21.06)	0.893	49.6 (30.26)	46.18 (28.64)	0.77	0.242	0.172	0.169	0.648
CD8 effector pSTAT3 (Mean)	38.18 (127.94)	4.44 (3.67)	0.191	2.79 (1.22)	7.99 (12.3)	0.168	4.06 (3.44)	3.29 (2.7)	0.343	0.248	0.166	0.84	0.148
BCells pNF-kB (Mean)	40.04 (19.4)	52.27 (27.6)	0.244	36.15 (14.35)	34.91 (12.59)	0.542	33.15 (17.9)	32.89 (17.89)	0.492	0.25	0.186	0.153	0.927
CD8 naive pSTAT3 (Mean)	5.1 (1.24)	7.43 (3.82)	0.048	6.04 (2.73)	6.77 (3.58)	0.191	4.7 (1.11)	5.26 (2.62)	1	0.251	0.131	0.362	0.376
CD4 αβ T cells (%Total T cells)	66.12 (7.6)	65.16 (7.52)	0.208	69.64 (8.27)	69.99 (7.85)	0.51	60.35 (6.89)	59.89 (8.26)	0.413	0.252	1	0.15	0.18
Neutrophils (% of CD45)	62.86 (9.47)	64.93 (11.9)	0.454	66.8 (5.74)	65.94 (6.87)	0.903	62.27 (8.52)	59.47 (7.81)	0.175	0.255	0.121	0.477	0.317
CD8 naive PD1 (Mean)	1.04 (0.87)	0.99 (0.69)	0.889	1.14 (1.08)	0.97 (0.73)	0.208	1.05 (0.8)	1.02 (0.42)	0.492	0.255	0.402	0.397	0.114
Total Monocytes TIGIT (Mean)	1.82 (1.68)	2.55 (1.8)	0.059	1.42 (0.89)	1.46 (1.19)	0.889	1.55 (1.05)	1.63 (0.93)	0.625	0.257	0.306	0.112	0.733
CD4 effector memory CD95 (Mean)	65.02 (20.58)	56.4 (19.01)	0.013	65.44 (11.42)	61.2 (18.95)	0.455	57.46 (13.04)	56.78 (16.16)	0.846	0.262	0.088	0.397	0.605
CD8 central memory pNF-kB (Mean)	64.15 (23.85)	75.66 (24.86)	0.021	60.46 (21.86)	61.86 (21.24)	0.685	58.25 (29.62)	62.11 (33.56)	1	0.264	0.154	0.2	0.832
CD4 naive TIGIT (Mean)	3.58 (2.53)	5.3 (2.83)	0.033	2.48 (1.09)	2.81 (1.56)	0.635	2.49 (1.1)	2.84 (1.32)	0.084	0.265	0.232	0.153	0.693
CD4 central memory CD95 (Mean)	52.44 (10.78)	49.27 (12.24)	0.094	51.24 (7.32)	48.75 (10.06)	0.34	51.04 (13.29)	51.88 (15.65)	0.846	0.267	0.145	0.555	0.239
CD4 effector memory pNF-kB (Mean)	73.83 (22.77)	80.25 (21.41)	0.127	71.16 (23.61)	69 (21.99)	0.542	66.69 (32.78)	69.43 (34.03)	0.77	0.271	0.42	0.186	0.239
CD4 effector memory (% of CD4+ αβT Cells)	9.52 (5.79)	11.87 (5.28)	0.015	8.31 (2.48)	9.79 (3.02)	0.153	10.78 (6.27)	11.57 (5.48)	0.365	0.276	0.148	0.252	0.687
Myeloid DC pS6 (Mean)	6.36 (1.49)	6.2 (1.4)	0.505	5.87 (2.19)	4.24 (1.35)	0.008	5.8 (2.61)	5.16 (2.34)	0.084	0.285	0.522	0.125	0.446
CD8αβ pSTAT3 (Mean)	4.85 (1.06)	6.26 (2.39)	0.094	5.46 (2.65)	6.29 (3.53)	0.244	4.47 (1.28)	4.7 (2.34)	0.492	0.287	0.172	0.457	0.264
CD4 (% of CD45)	12.49 (5.23)	11.14 (4.81)	0.599	11 (3.66)	11.49 (3.71)	0.675	11.1 (3.39)	12.07 (4.38)	0.413	0.311	0.164	0.29	0.687
CD8 effector CD38 (Mean)	7.22 (10.71)	6.99 (4.5)	0.107	5.24 (2.74)	5.7 (3.66)	1	5.08 (2.65)	5.04 (2.01)	0.919	0.313	0.164	0.252	0.936
B Cells (% of CD45)	2.54 (1.27)	2.3 (1.27)	0.286	2.51 (0.84)	2.78 (1.28)	0.903	2.78 (1.06)	3.28 (1.54)	0.413	0.327	0.132	0.371	0.727
B Cells pSTAT3 (Mean)	3.34 (1.2)	4.16 (1.49)	0.068	2.97 (1.56)	3.05 (1.39)	1	2.33 (0.79)	2.78 (1.12)	0.232	0.332	0.605	0.169	0.376
CD4 naive pSTAT3 (Mean)	6.44 (1.15)	6.82 (1.27)	0.497	7.99 (2.63)	8.39 (3.19)	0.588	6.6 (1.68)	5.94 (1.77)	0.193	0.339	0.257	0.626	0.208
CD8 effector memory pS6 (Mean)	4.42 (3.33)	3.51 (0.85)	0.787	3.02 (0.83)	2.45 (0.74)	0.022	3.12 (1.49)	2.89 (1.37)	0.236	0.339	0.343	0.243	0.343
Total Monocytes HLA.DR (Mean)	127.73 (61.32)	113.07 (50.58)	0.061	112.47 (51.35)	100.01 (36.86)	0.268	113.65 (39.6)	114.16 (41.38)	0.831	0.349	0.126	0.715	0.434
Total T cells (% CD45)	18.59 (6.21)	16.99 (6.57)	0.639	15.7 (4.67)	16.42 (4.65)	0.626	18.52 (5.49)	19.98 (5.84)	0.175	0.353	0.169	0.4	0.572
CD8αβ T cells CD38 (Mean)	10.02 (4.37)	9.98 (4.12)	0.934	9.87 (3.42)	9.16 (3.6)	0.173	9.42 (4.22)	9.6 (4.3)	0.465	0.368	0.76	0.331	0.183
CD8 effector (% of CD8+ αβT Cells)	8.22 (7.22)	10.04 (7.74)	0.03	5.09 (4.78)	7.01 (6.19)	0.173	10.75 (11.17)	10.06 (9.75)	0.465	0.371	0.164	0.377	0.809
CD8ab TIGIT (Mean)	14.61 (6.45)	13.73 (5.94)	0.455	12.42 (5.35)	11.71 (5.21)	0.146	14.49 (6.25)	15.29 (7.21)	0.432	0.383	0.257	0.84	0.232
CD8 naive CD95 (Mean)	8.18 (2.44)	9.12 (3.46)	0.376	8.07 (2.55)	7.74 (1.97)	0.893	7.49 (3.46)	6.72 (1.95)	0.557	0.393	0.186	0.418	0.664
non-classical monocytes TIGIT (Mean)	2.61 (1.95)	3.57 (2.68)	0.273	1.86 (1.34)	1.89 (1.4)	0.6	2.12 (1.41)	2.06 (1.13)	1	0.397	0.208	0.362	0.738
CD8αβ Tcells (% of Total T Cells)	25.69 (8.04)	25.77 (7.95)	0.776	23.22 (6.8)	22.59 (6.74)	0.052	28.54 (7.36)	28.49 (9.26)	0.894	0.401	0.736	0.132	0.681
CD4 effector memory pSTAT3 (Mean)	5.1 (0.94)	5.9 (1.56)	0.146	5.93 (1.96)	7.87 (4.17)	0.1	5.18 (1.27)	5.48 (2.14)	1	0.405	0.483	0.418	0.239
CD4αβ pSTAT3 (Mean)	6.28 (0.99)	6.75 (1.26)	0.414	7.36 (2.23)	7.75 (2.81)	0.497	6.41 (1.71)	5.97 (1.38)	0.375	0.407	0.284	0.724	0.257
CD4αβ pS6 (Mean)	5.28 (2.76)	4.51 (0.9)	0.635	4.89 (2.08)	3.67 (1.36)	0.04	3.86 (1.89)	3.49 (1.68)	0.084	0.409	0.577	0.311	0.257
Classical monocytes HLA.DR (Mean)	106.7 (60.91)	91.38 (48.55)	0.02	91.72 (48.29)	79.81 (32.89)	0.296	91.85 (40.56)	89.07 (40.24)	0.638	0.417	0.164	0.621	0.572
Myeloid DC PD1 (Mean)	0.4 (0.09)	0.43 (0.12)	0.463	0.39 (0.22)	0.38 (0.21)	0.727	0.29 (0.07)	0.32 (0.08)	0.202	0.418	0.926	0.412	0.172
Myeloid DC CD38 (Mean)	115.07 (29.32)	109.07 (24.79)	0.164	107.17 (25.65)	101.37 (26.6)	0.683	91.98 (21.1)	93.56 (17.84)	0.765	0.424	0.185	0.616	0.501
CD8 central memory (% of CD8+ αβT Cells)	27.9 (10.21)	25.83 (9.71)	0.121	28.12 (12.16)	26.46 (11.49)	0.091	24.56 (13.87)	25.97 (12.48)	0.689	0.428	0.243	1	0.286
CD8 effector memory TIGIT (Mean)	17.02 (8.92)	18.25 (8.32)	0.311	19.65 (7.22)	18.44 (6.62)	0.376	20.19 (9.85)	19.89 (10.02)	0.846	0.429	0.483	0.204	0.738
CD8 central memory TIGIT (Mean)	24.48 (9.23)	22.38 (8.21)	0.305	26.06 (7.56)	24.19 (5.07)	0.127	25.65 (8.77)	25.46 (9.09)	0.846	0.435	0.292	0.92	0.257
CD8 effector memory CD38 (Mean)	4.95 (3.7)	6.77 (6.92)	0.489	4.38 (1.83)	4.48 (2.21)	0.944	4.14 (3.02)	3.67 (2.24)	0.278	0.448	0.281	0.621	0.344
classical monocytes CD38 (Mean)	81.21 (25.6)	75.49 (21.2)	0.041	78.34 (16.4)	74.64 (18.55)	0.463	69.34 (20.73)	69.65 (19.28)	0.919	0.457	0.217	0.561	0.572
Natural Killer (% of CD45)	2.27 (1.18)	2.12 (1.64)	0.107	1.95 (0.95)	1.89 (0.75)	0.808	2.41 (1.01)	2.64 (1.15)	0.765	0.46	0.281	0.359	0.727
T-cells pSTAT3 (Mean)	5.86 (1.06)	6.72 (1.51)	0.17	6.73 (2.13)	7.35 (2.89)	0.34	5.71 (1.48)	5.64 (1.77)	0.695	0.464	0.226	1	0.376
B-cells pS6 (Mean)	25.62 (19.19)	16.08 (5.35)	0.036	19.77 (8.9)	15.66 (10.73)	0.064	16.75 (10.52)	14.48 (8.31)	0.322	0.484	0.203	0.608	0.648
non-classical monocytes (% of Total Monocy	10.82 (4.05)	8.98 (5.58)	0.107	12.99 (5.84)	11.97 (5.44)	0.426	12.38 (3.81)	10.26 (4.14)	0.014	0.491	0.838	0.354	0.291
CD8 effector TIGIT (Mean)	30.2 (17.43)	22.9 (11.36)	0.013	25.73 (9.38)	20.89 (10.27)	0.08	33.57 (25.56)	31.05 (23.61)	0.232	0.496	0.257	0.545	0.605
Total NK CD38 (Mean)	137.64 (48.09)	142.24 (38.95)	0.804	138.09 (54.13)	131.46 (44.97)	0.426	105.69 (49)	108.54 (47.73)	0.765	0.501	0.897	0.477	0.222
Early NKs PD1 (Mean)	0.26 (0.09)	0.29 (0.13)	0.65	0.29 (0.14)	0.26 (0.07)	0.583	0.18 (0.04)	0.21 (0.06)	0.26	0.504	1	0.442	0.238
CD4 effector pNF-kB (Mean)	67.62 (26.28)	73.79 (34.57)	0.701	59.66 (25)	55.01 (24.59)	0.221	53.32 (35.68)	51.31 (26.29)	0.77	0.506	0.446	0.311	0.648
Transitional Monocytes HLA.DR (Mean)	346.13 (108.05)	345.07 (104.69)	0.934	284.57 (104.26)	279.58 (115.32)	0.903	330.45 (131.34)	360 (109.05)	0.056	0.506	0.324	0.965	0.311
B cells TIGIT (Mean)	6.65 (5.39)	8.1 (4.08)	0.414	2.36 (1.54)	3.06 (2.82)	0.787	3.1 (2.11)	2.74 (1.79)	0.432	0.507	0.313	0.7	0.385
CD4 effector CD95 (Mean)	38.83 (30.2)	23.98 (17.12)	0.022	29.99 (22.38)	24.81 (21.62)	0.305	23.89 (24.35)	18.96 (15.06)	0.275	0.514	0.313	0.39	0.879
Total Monocytes CD38 (Mean)	71.7 (22.92)	68.65 (19.39)	0.359	67.72 (17.33)	64.9 (16.72)	0.855	60.98 (19.78)	63.07 (18.38)	0.577	0.514	0.259	0.747	0.477
non-classical monocytes PD1 (Mean)	0.33 (0.13)	0.37 (0.12)	0.311	0.29 (0.13)	0.27 (0.08)	0.806	0.25 (0.07)	0.27 (0.08)	0.406	0.521	0.78	0.33	0.385
Late NKs TIGIT (Mean)	15.38 (5.93)	14.12 (3.93)	0.168	14.43 (4.78)	13.33 (3.58)	0.069	16.87 (9.31)	14.73 (7.99)	0.014	0.535	0.376	0.758	0.336
Total memory B Cells HLA.DR (Mean)	357.87 (157.02)	314.33 (119.91)	0.005	367.5 (95.69)	337.93 (73.67)	0.187	322.55 (57.47)	314.82 (56.73)	0.465	0.547	0.35	0.513	0.511
CD8 (% of CD45)	4.65 (1.72)	4.36 (2.22)	0.776	3.65 (1.46)	3.74 (1.58)	0.808	5.31 (2.18)	5.58 (2.15)	0.248	0.561	0.281	0.78	0.536
CD8 effector memory pNF-kB (Mean)	62.83 (22.65)	69.89 (22.81)	0.146	59.39 (23.25)	59.98 (22.32)	0.946	54.15 (28.24)	57.13 (30.24)	0.922	0.562	0.376	0.39	0.879
Early NKs CD38 (Mean)	158.72 (54.8)	160.09 (44.63)	0.955	153.32 (60.77)	144.84 (49.94)	0.296	121.09 (58.65)	122.37 (56.35)	0.76	0.564	0.856	0.471	0.298
CD4 naive pS6 (Mean)	8.46 (7.15)	6.12 (1.7)	0.244	7.16 (3.33)	5.34 (3)	0.068	4.91 (2.78)	4.25 (2.23)	0.16	0.568	0.832	0.545	0.284
CD4αβ CD95 (Mean)	34.37 (8.27)	33.05 (8.57)	0.142	32.33 (7.06)	30.96 (8.77)	0.305	34.04 (14.15)	34.22 (13.77)	0.846	0.581	0.336	0.878	0.438
non-classical monocytes CD95 (Mean)	20.97 (7.49)	20.88 (6.3)	0.906	21.38 (5.79)	20.21 (6.09)	0.414	18.3 (5.39)	19.14 (6.64)	0.625	0.591	0.535	0.687	0.343
non-classical monocytes pS6 (Mean)	6.87 (2.81)	5.69 (1.31)	0.147	6.23 (1.78)	4.95 (2.19)	0.017	5.65 (3.03)	5.09 (2.48)	0.232	0.597	1	0.59	0.257
Myeloid dc CD95 (Mean)	29.48 (6.24)	29.86 (6.68)	0.893	30.62 (6.07)	29.79 (5.62)	0.735	26.41 (6.1)	28.15 (6.67)	0.492	0.613	0.563	0.65	0.376
Total monos CD95 (Mean)	47.69 (12.76)	47.92 (11.35)	1	48.55 (9.59)	47.57 (8.84)	0.972	44.95 (11.73)	48.41 (13.48)	0.322	0.616	0.446	0.88	0.385
B Cells HLA.DR (Mean)	356 (158.46)	313 (117.79)	0.015	367.57 (94.41)	334.43 (75.11)	0.116	325.91 (58.31)	317.73 (57.64)	0.52	0.62	0.392	0.76	0.46
classical monocytes pSTAT3 (Mean)	15.72 (4.5)	14.81 (3.24)	0.455	18.88 (4.95)	16.9 (5.22)	0.244	15.61 (3.63)	15.45 (2.9)	1	0.622	0.664	0.65	0.343
CD4 effector memory TIGIT (Mean)	4.53 (2.23)	5.78 (2.6)	0.003	4.96 (2.15)	5.14 (2.27)	0.484	4.73 (2.47)	5.48 (2.66)	0.16	0.629	0.976	0.259	0.733
CD8 central memory CD95 (Mean)	41.21 (10.35)	38.69 (10.55)	0.127	39.54 (7.49)	37.69 (8.66)	0.279	36.07 (12.7)	34.74 (12.55)	0.153	0.631	0.385	0.479	1
Naïve B cells HLA.DR (Mean)	354.93 (161.09)	315.87 (128.52)	0.027	369 (93.98)	332.57 (75.85)	0.09	330.09 (59.4)	320.73 (59.07)	0.35	0.633	0.452	1	0.381
Transitional Monocytes (% of Total Monocyte	3.65 (1.54)	3.26 (1.49)	0.551	4.32 (1.45)	3.56 (1.76)	0.068	3.23 (0.91)	2.95 (0.91)	0.638	0.641	0.876	0.419	0.467
CD4 central memory pSTAT3 (Mean)	6.28 (1.25)	6.62 (1.62)	0.893	7 (2.07)	7.13 (2.32)	0.839	6.62 (2.19)	5.91 (1.09)	0.432	0.646	0.495	0.801	0.41
CD8 effector memory (% of CD8+ αβT Cells)	6.56 (6.12)	8.19 (4.76)	0.107	8.09 (7.52)	9.2 (6.96)	0.173	9.17 (6.72)	10 (5.42)	0.638	0.652	0.443	0.541	0.647
Total Monos (% of CD45)	5.56 (1.72)	5.29 (2.29)	0.561	5.83 (1.62)	5.42 (1.48)	0.241	5.51 (1.15)	5.66 (0.95)	0.594	0.67	0.452	0.983	0.443
Total Monos pSTAT3 (Mean)	14.2 (4)	13.78 (2.87)	0.889	16.58 (4.39)	15.11 (4.52)	0.376	13.89 (3.27)	13.92 (2.24)	0.922	0.675	0.951	0.608	0.352
Tcells CD95 (Mean)	30.22 (8.01)	29.22 (7.84)	0.216	28.54 (6.16)	27.45 (7.42)	0.208	28.55 (11.32)	28.44 (10.77)	0.636	0.698	0.605	0.918	0.368
Tcells pS6 (Mean)	7.46 (7.03)	5.26 (1.14)	0.216	6.34 (3.36)	4.74 (2.46)	0.094	4.5 (2.26)	3.97 (1.96)	0.105	0.701	1	0.545	0.446
Total Monos PD1 (Mean)	0.55 (0.09)	0.57 (0.14)	0.432	0.5 (0.16)	0.48 (0.12)	0.753	0.44 (0.1)	0.45 (0.1)	0.678	0.701	0.951	0.473	0.522
Late NKs CD38 (Mean)	122.05 (46.32)	124.91 (34.64)	0.932	122.44 (51.93)	116.65 (43.74)	0.542	91.99 (46.34)	95.48 (43.69)	0.413	0.701	0.755	0.662	0.403
Tcells TIGIT (Mean)	9.26 (4.4)	9.59 (3.74)	0.542	7.84 (2.55)	7.77 (2.38)	1	8.63 (3.24)	9.25 (3.91)	0.432	0.705	0.71	0.724	0.41
CD8 naive TIGIT (Mean)	7.97 (4.71)	8.07 (4.75)	0.839	5.42 (3.47)	5.05 (3.14)	0.376	7.09 (4.05)	7.13 (4.13)	0.625	0.709	0.901	0.489	0.522
CD8 effector memory pSTAT3 (Mean)	5.62 (6.68)	6.05 (6.77)	0.244	4.67 (3.88)	5.61 (5.51)	1	3.66 (3.01)	4.41 (4.2)	0.492	0.715	0.563	0.579	0.522
CD8 effector memory PD1 (Mean)	7.33 (3.72)	7.08 (3.14)	0.906	11.06 (8.34)	10.04 (5.64)	0.839	9.99 (5.59)	10.27 (5.65)	0.625	0.732	0.693	0.762	0.446
Classical monocytes (% of Total Monocytes)	82.17 (5.51)	84.39 (7.22)	0.151	79.76 (6.79)	80.52 (6.98)	0.583	80.63 (5.04)	83.7 (4.78)	0.097	0.745	0.959	0.499	0.565
CD4 central memory (% of CD4+ αβT Cells)	47.97 (10.41)	46.37 (9.25)	0.121	47.76 (10.82)	45.95 (11.45)	0.209	45.5 (11.34)	44.82 (11.34)	0.689	0.767	0.483	1	0.603
Late NKs PD1 (Mean)	0.21 (0.07)	0.24 (0.11)	0.239	0.22 (0.09)	0.23 (0.1)	0.529	0.18 (0.04)	0.21 (0.03)	0.102	0.777	0.709	0.739	0.513
CD8 effector memory CD95 (Mean)	40.76 (15.13)	39.16 (15.45)	0.542	36.65 (8.26)	36.66 (10.38)	0.839	31.17 (13.81)	30.18 (13.33)	0.124	0.784	0.976	0.614	0.535
MAIT/NKT CD38 (Mean)	4.34 (3.65)	4.42 (2.5)	0.89	3.09 (1.27)	2.92 (1.67)	0.542	2.74 (1.3)	2.4 (1.49)	0.175	0.785	0.474	0.813	0.767
Late Nks pSTAT3 (Mean)	2.73 (0.99)	3.38 (1.4)	0.168	3.64 (3.08)	3.25 (1.78)	0.787	2.41 (0.92)	2.49 (0.88)	0.557	0.795	0.605	0.579	0.976
CD4 central memory TIGIT (Mean)	10.61 (4.27)	10.69 (3.45)	0.906	10.76 (2.99)	10.76 (2.92)	1	9.85 (2.99)	10.33 (3.23)	0.557	0.796	0.605	0.959	0.556
non-classical monocytes pSTAT3 (Mean)	5.52 (1.18)	5.93 (0.83)	0.414	5.84 (2)	6.32 (1.85)	0.944	5.53 (1.78)	5.39 (1.34)	0.922	0.821	0.522	0.918	0.733
Early NKs TIGIT (Mean)	25.09 (6.66)	22.58 (4.63)	0.057	23.83 (9.79)	22.04 (6.56)	0.244	25.53 (13.03)	23.2 (10.87)	0.027	0.83	0.976	0.608	0.648
Classical monocytes PD1 (Mean)	0.59 (0.09)	0.61 (0.18)	0.844	0.54 (0.16)	0.52 (0.12)	0.505	0.48 (0.1)	0.48 (0.1)	0.798	0.846	0.901	0.739	0.576
CD8 effector pS6 (Mean)	14.18 (16.66)	8.5 (3.35)	0.11	11.75 (10.96)	8.35 (6.29)	0.216	7.24 (5.06)	5.98 (3.26)	0.232	0.859	0.563	0.778	0.976
CD8 naive pS6 (Mean)	16.87 (16.46)	9.42 (3.89)	0.08	14.32 (8.7)	10.42 (7.25)	0.273	8.46 (5.65)	6.91 (4.25)	0.193	0.859	0.693	0.65	0.927
CD4ab TIGIT (Mean)	7.26 (3.7)	8.01 (3.31)	0.126	6.66 (2.24)	6.79 (2.15)	0.735	6.22 (2.24)	6.67 (2.41)	0.193	0.862	0.832	0.65	0.738
CD8 effector PD1 (Mean)	2.42 (2.15)	1.87 (1.05)	0.497	4.3 (4.88)	3.09 (2.78)	0.244	3.04 (2.8)	2.58 (1.68)	0.636	0.868	0.832	0.724	0.664
CD8ab pS6 (Mean)	11.91 (12.36)	6.97 (1.88)	0.057	10.72 (7.62)	7.71 (5.34)	0.216	6.04 (3.39)	5.08 (2.69)	0.105	0.87	0.605	0.762	0.976
Early NKs pS6 (Mean)	10.21 (8.05)	6.85 (2.1)	0.127	8.44 (4.09)	5.54 (2.75)	0.017	6.96 (4.19)	5.07 (2.74)	0.084	0.873	0.738	0.65	0.927
CD8 effector CD95 (Mean)	16.18 (5.19)	15.33 (5.05)	0.279	19.24 (5.39)	17.5 (6.73)	0.093	15.43 (4.7)	14.56 (4.58)	0.126	0.874	0.852	0.663	0.733
BCells PD1 (Mean)	0.23 (0.13)	0.24 (0.1)	0.789	0.24 (0.18)	0.2 (0.06)	1	0.18 (0.07)	0.17 (0.04)	0.943	0.882	0.95	0.681	0.708
CD4 naïve (% of CD4+ αβT Cells)	40.85 (12.04)	39.06 (11.6)	0.277	42.76 (11.69)	41.64 (10.45)	0.572	42.01 (13.66)	41.4 (13.56)	0.638	0.882	0.659	0.76	0.848
Myeloid DC (% of CD45)	0.15 (0.07)	0.16 (0.06)	0.442	0.18 (0.13)	0.18 (0.1)	0.646	0.2 (0.09)	0.21 (0.11)	0.441	0.924	0.716	1	0.783
CD8ab CD95 (Mean)	20.18 (6.72)	19.79 (6.55)	0.414	19.2 (6.8)	18.25 (6.39)	0.376	18.46 (8.15)	18.18 (7.79)	0.625	0.933	0.975	0.898	0.648
CD8 central memory CD38 (Mean)	11.58 (9.65)	10.75 (7.56)	0.978	10.19 (4.58)	9.15 (4.89)	0.463	8.16 (4.42)	8.12 (3.35)	0.898	0.941	0.959	0.813	0.767
Eosinophils (% of CD45)	2.54 (1.84)	2.97 (2.87)	0.934	3.28 (2.07)	3.04 (2.35)	0.391	2.48 (1.46)	2.25 (1.2)	0.577	0.946	0.76	0.847	0.979
Late NKs pS6 (Mean)	9.77 (7.83)	6.3 (2.04)	0.068	7.59 (3.32)	5.51 (2.59)	0.048	6.13 (3.62)	5.01 (2.71)	0.084	0.97	0.738	0.92	0.976
CD8 naïve (% Total CD8)	57.32 (15.81)	55.95 (14.75)	0.268	58.69 (21.09)	57.31 (19.09)	0.761	55.53 (19.8)	53.95 (17.98)	0.638	0.976	0.917	0.896	0.851
CD4 effector pSTAT3 (Mean)	58.83 (183.13)	16.61 (35.99)	0.685	5.91 (1.88)	8.05 (8.72)	1	6.08 (4.09)	4.85 (1.53)	0.77	0.977	0.976	0.801	0.976
CD4 effector TIGIT (Mean)	6.96 (4.28)	8.87 (6.07)	0.735	5.26 (3.32)	6.81 (6.8)	0.685	5.49 (4.97)	5.75 (5.44)	0.557	0.977	1	0.88	0.879
Total Memory B Cells (% Total B cells)	52.04 (6.27)	50.35 (9.14)	0.14	46.31 (5.21)	44.44 (5.44)	0.194	53.25 (9.84)	51.13 (10.68)	0.102	0.983	0.76	0.983	0.979
Myeloid DC pSTAT3 (Mean)	8.29 (1.93)	9.05 (5.05)	0.839	10.31 (3.24)	9.99 (3.64)	0.787	8.4 (2.27)	8.12 (1.45)	0.846	0.992	0.927	0.96	0.927
Naive B Cells (% Total B Cells)	47.49 (6.36)	49.15 (9.8)	0.135	53.29 (5.2)	55.36 (5.51)	0.153	46.53 (9.94)	48.7 (10.76)	0.083	0.992	0.878	0.983	0.956

P-Value is from paired Wilcoxon Test. Overall is p-value from Kruskal Wallis Test, *Between Group Comparisons* were done with Wilcoxon's Test

Supplementary Table 5. Changes in circulating immune cells assessed by cytometry by time-of-flight (CyTOF)
